# Transcriptome-informed metabolic modeling reveals astrocyte-specific vulnerabilities in mild cognitive impairment and Alzheimer’s disease progression

**DOI:** 10.3389/fbinf.2026.1816121

**Published:** 2026-06-02

**Authors:** Andrea Angarita-Rodríguez, Viviana Vargas-López, Andrés Pinzón, Adrián Sandoval-Hernandez, Kai Kang, Leping Li, Jason Papin, Pedro Puentes-Rozo, Andrés Felipe Aristizábal, Janneth González

**Affiliations:** 1 Departamento de Nutrición y Bioquímica, Facultad de Ciencias, Pontificia Universidad Javeriana, Bogotá, Colombia; 2 Laboratorio de Bioinformática y Biología de Sistemas, Universidad Nacional de Colombia Bogotá, Bogotá, Colombia; 3 Biostatistics and Computational Biology Branch at NIEHS, Bethesda, MD, United States; 4 Grupo de Neurociencias y Muerte Celular, Instituto de Genética, Universidad Nacional de Colombia, Bogotá, Colombia; 5 Departamento de Química, Facultad de Ciencias, Universidad Nacional de Colombia, Bogotá, Colombia; 6 Department of Mathematics and Statistics, University of North Carolina Wilmington, Wilmington, NC, United States; 7 Department of Biomedical Engineering, University of Virginia, Charlottesville, VA, United States; 8 Department of Medicine, Division of Infectious Diseases and International Health, University of Virginia, Charlottesville, VA, United States; 9 Department of Biochemistry and Molecular Genetics, University of Virginia, Charlottesville, VA, United States; 10 Grupo de Neurociencias del Caribe, Unidad de Neurociencias Cognitivas, Universidad Simón Bolívar, Barranquilla, Colombia; 11 Grupo de Neurociencias del Caribe, Universidad del Atlántico, Barranquilla, Colombia

**Keywords:** genome-scale metabolic models, flux balance analysis, metabolic reprogramming, transcriptome, deconvolution, mild cognitive impairment, Alzheimer’s disease, astrocyte

## Abstract

**Introduction:**

Astrocytes are essential for maintaining neuronal homeostasis, yet their stage-specific contribution to mild cognitive impairment (MCI) and Alzheimer’s disease (AD) remains insufficiently understood. This study aimed to investigate astrocyte-associated transcriptional and metabolic alterations across the control-MCI-AD continuum using integrated transcriptomic and genome-scale metabolic modeling approaches.

**Methods:**

Transcriptomic profiles from hippocampal CA1 tissue (GSE28146) were analyzed across four clinical conditions (control, early MCI, advanced MCI, and AD). Astrocyte-associated expression programs were inferred using the unsupervised deconvolution algorithm CDSeq and validated through canonical marker enrichment, correlation with external reference signatures, and comparison with an independent single-nucleus RNA-seq astrocyte pseudobulk dataset. The inferred profiles were integrated into a curated human astrocyte genome-scale metabolic model to generate condition-specific models, which were analyzed using flux balance analysis (FBA) and flux variability analysis (FVA).

**Results:**

The analyses supported stage-dependent remodeling of astrocyte-associated transcriptional and metabolic programs during disease progression. Early MCI was associated with signaling and stress-adaptation changes, whereas advanced MCI and AD showed broader disruption of synaptic support, redox homeostasis, and inflammatory-related programs. Model predictions indicated a progressive reduction in a biomass-derived maintenance proxy from control to advanced MCI, followed by a partial rebound in AD, suggesting a compensatory shift toward reactive-like astrocyte states rather than full functional recovery. Flux variability analysis revealed reduced metabolic flexibility across disease stages, particularly in glutamate-glutamine cycling, glutathione/redox metabolism, glycolysis-pyruvate metabolism, cholesterol handling, and one-carbon/folate metabolism.

**Discussion:**

These findings support the view that astrocytes undergo progressive, stage-specific metabolic reprogramming during the transition from healthy aging to AD. Early alterations in redox regulation, neurotransmitter cycling, and mitochondrial function may contribute to early neuronal vulnerability. This work highlights astrocyte-centered pathways as potential targets for future experimental validation and therapeutic exploration.

## Graphical Abstract

**Figure:**
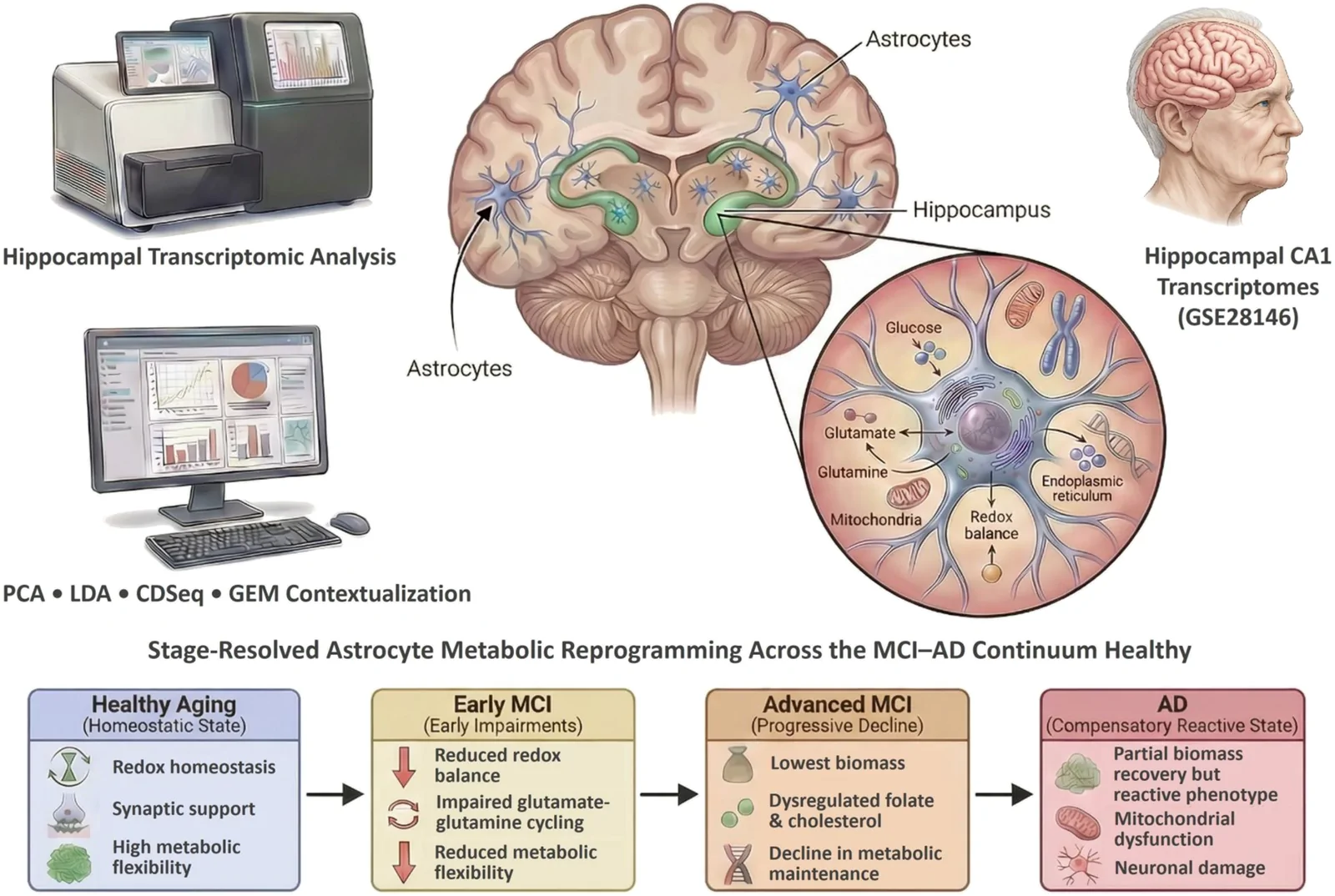

## 1 Introduction

Neurodegenerative diseases (NDs), including AD, represent a major global health challenge due to their progressive and irreversible impact on cognitive function and quality of life (Ping et al., 2020). Mild Cognitive Impairment (MCI) is widely recognized as an intermediate clinical state between normal aging and dementia, characterized by measurable cognitive decline that does not yet meet criteria for dementia (Fornazzari, 2001; Mufson et al., 2012). Importantly, MCI is a heterogeneous condition with a substantial proportion of individuals progressing to AD (Ahmed et al., 2008), while others remain stable or revert to cognitively normal stages (Dunne et al., 2021). Understanding the biological mechanisms underlying this transitional stage is therefore critical for identifying early pathological events and potential intervention targets (Table 1).

**TABLE 1:** Main metabolite groups involved in the astrocytic biomass maintenance reaction. Each group contributes to essential cellular functions such as neurotransmitter cycling, membrane integrity, redox balance, and energy production. These metabolites reflect the metabolic demands required to sustain astrocyte viability and their supportive role in the central nervous system.

Metabolite group	Key biological Function in astrocytes
Essential and non-essential amino acids	Protein synthesis, neurotransmitter production, and regulation of the glutamate–glutamine cycle
Nucleotides (ATP, GTP, *etc.*)	Provide energy for active transport, cellular repair, and signaling
Membrane lipids (phospholipids, cholesterol)	Maintain membrane integrity and mediate cholesterol transport to neurons
Glutathione (GSH)	Antioxidant defense against reactive oxygen species (ROS)
Neurotransmitter precursors	Regulation of GABAergic metabolism and support of synaptic function
Cofactors (FAD, NAD^+^, *etc.*)	Redox balance and mitochondrial enzymatic activity

Traditionally, neurodegenerative research has focused primarily on neuronal dysfunction and loss. However, accumulating evidence indicates that neurodegeneration arises from complex, multicellular processes involving dynamic interactions among neurons, astrocytes, microglia, oligodendrocytes, and vascular cells (Allen and Eroglu, 2017; Farhy-tselnicker & Allen, 2018; Shan et al., 2021). Among these, astrocytes have emerged as key regulators of brain homeostasis due to their central roles in energy metabolism, neurotransmitter recycling, redox balance, synaptic modulation, and neurovascular coupling (Saint-Martin and Goda, 2023; Volterra and Meldolesi, 2005). Astrocytic dysfunction has been increasingly implicated in the early stages of neurodegenerative diseases, including AD, where alterations in astrocyte metabolism, inflammatory signaling, and lipid handling precede or accompany neuronal damage (Siracusa et al., 2019; Sofroniew, 2014).

A hallmark of astrocyte involvement in neurodegeneration is reactive astrogliosis, a phenotypic transformation characterized by profound changes in morphology, gene expression, and metabolism (Giovannoni and Quintana, 2021; Li et al., 2019). While reactive astrocytes can exert neuroprotective functions under certain conditions, sustained or maladaptive activation has been associated with impaired neurotransmitter clearance, oxidative stress, metabolic inflexibility, and synaptic dysfunction (Wade et al., 2011). In the context of MCI, astrocytes are thought to undergo early functional alterations that compromise neuronal support, yet the specific metabolic consequences of these changes remain incompletely understood.

Transcriptomic studies have provided valuable insights into molecular alterations associated with MCI and AD, revealing dysregulation of pathways related to synaptic transmission, inflammation, mitochondrial function, and lipid metabolism (Huang et al., 2025; Park et al., 2024). More recently, single-cell and single-nucleus RNA sequencing approaches have identified disease-associated astrocyte states and reactive phenotypes in AD brains (Galea et al., 2022; Matusova et al., 2023). However, most transcriptomic analyses remain largely descriptive, and gene expression changes are often difficult to interpret in terms of their direct functional or metabolic consequences at the cellular level.

Genome-scale metabolic models (GEMs) provide a complementary framework to interpret omics data from a functional and mechanistic perspective. GEMs allow the simulation of metabolic fluxes under different physiological or pathological constraints, thereby revealing key metabolic pathways, bottlenecks, and potential therapeutic targets (Nielsen, 2017b; Orth et al., 2010). When informed with transcriptomic data, GEMs can be contextualized to reflect the metabolic state of specific cell types, enabling hypothesis-driven simulation and comparison across disease stages (Angarita-Rodríguez et al., 2024; Nielsen, 2017b). Constraint-based modeling approaches such as FBA and FVA enable the systematic exploration of metabolic capabilities, bottlenecks, and flexibility under defined conditions (Gianchandani et al., 2010; Ruppin et al., 2010). While GEMs have been widely applied in cancer biology and microbial systems, their application to brain-specific cell types remains comparatively limited (Nielsen, 2017b; 2017a). Existing metabolic modeling studies in neurodegeneration have largely focused on generic brain reconstructions, often without incorporating cell-type-specific or disease-stage-resolved transcriptomic information (Angarita-Rodríguez et al., 2024; González et al., 2020; Osorio et al., 2020).

While GEMs have been increasingly applied to study metabolic dysfunction in neurodegenerative diseases, their use has been largely restricted to neuronal or generic brain reconstructions. Existing astrocyte metabolic models have primarily been developed in controlled experimental settings to investigate specific metabolic perturbations, such as lipotoxicity, oxidative stress, or nutrient overload, rather than clinical disease progression (Angarita-Rodríguez et al., 2022; Osorio et al., 2020). To date, these astrocyte-focused models have not been systematically linked to patient-derived transcriptomic data nor applied to defined stages along the MCI-AD continuum.

To address these limitations, deconvolution methods have been developed to extract cell-type-specific transcriptional profiles from bulk tissue data (Kang et al., 2021). Unsupervised approaches such as CDSeq allow simultaneous estimation of cell-type proportions and expression profiles without requiring predefined reference signatures, making them particularly suitable for complex tissues such as the human brain (Im and Kim, 2023; Kang et al., 2021). When combined with GEMs, deconvolution-derived transcriptomic profiles offer a powerful strategy to generate context-specific metabolic models that reflect the functional state of individual cell types under pathological conditions.

In contrast to previous transcriptomic or metabolic modeling studies, the present work uniquely integrates unsupervised astrocyte-specific transcriptomic deconvolution with genome-scale metabolic modeling to investigate metabolic reprogramming across well-defined stages of cognitive decline. By contextualizing a curated human astrocyte GEM with deconvolved transcriptomic profiles from hippocampal CA1 tissue, we generate stage-resolved metabolic models representing control, early MCI, advanced MCI, and AD conditions. Using flux balance and flux variability analyses, we extend descriptive gene expression analyses by estimating condition-specific metabolic performance and flexibility in astrocytes along the MCI–AD continuum.

Through this integrative framework, we examine predicted early astrocyte-specific metabolic alterations affecting redox balance, neurotransmitter cycling, lipid metabolism, and mitochondrial function, providing hypothesis-generating insights into how astrocytic dysfunction may relate to neuronal vulnerability during prodromal stages of AD. These findings suggest that astrocyte metabolism may represent a relevant and potentially targetable axis in early neurodegenerative progression.

## 2 Materials and methods

**FIGURE 1:**
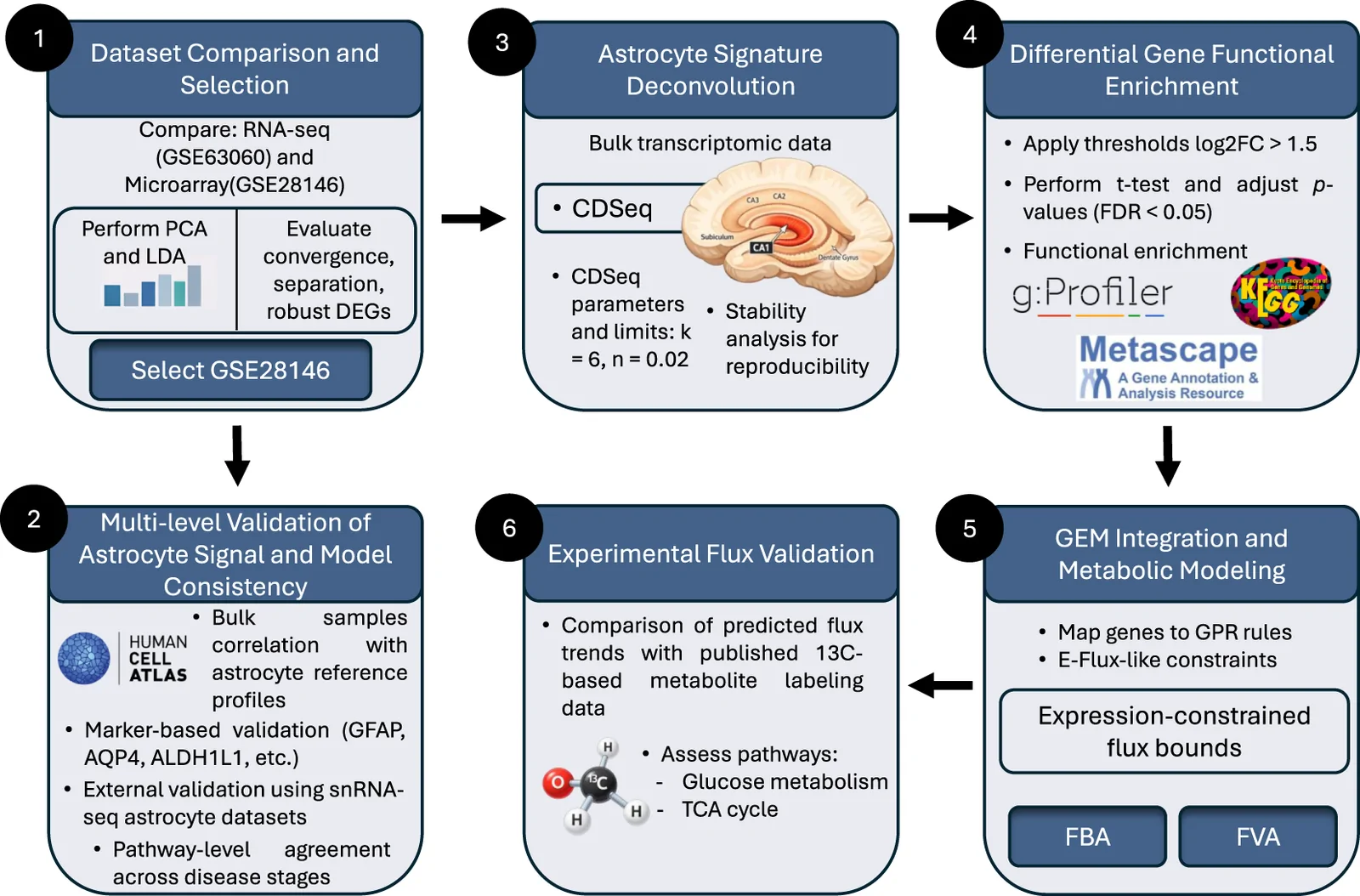
Schematic overview of the transcriptome-informed metabolic modeling workflow used to identify astrocyte-specific vulnerabilities in MCI. The pipeline comprises seven main stages: (1) dataset comparison and selection, including evaluation of RNA-seq (GSE63060) and microarray (GSE28146) data using PCA and LDA to ensure robust separation and convergence; (2) multi-level validation of astrocyte signals through correlation with reference profiles, marker-based validation (e.g., GFAP, AQP4, ALDH1L1), and external single-nucleus RNA-seq datasets; (3) deconvolution of astrocyte-specific transcriptomic signatures from bulk data using CDSeq, including stability assessment; (4) differential gene expression and functional enrichment analysis using statistical thresholds (log2FC > 1.5, FDR <0.05); (5) integration of expression data into a genome-scale metabolic model (GEM) of human astrocytes using GPR rules and expression-constrained (E-Flux-like) bounds, followed by flux balance analysis (FBA) and flux variability analysis (FVA); and (6–7) validation of predicted metabolic fluxes through comparison with experimental data, including published ^13C-based metabolite labeling studies, to assess consistency in key pathways such as glucose metabolism and the TCA cycle.

We implemented a transcriptome-to-metabolism computational pipeline to investigate astrocyte-specific metabolic alterations in MCI and AD. Starting from hippocampal transcriptomic data, we performed preprocessing, dataset selection, differential expression and enrichment analysis. Astrocyte-associated expression signals were inferred *via* unsupervised deconvolution, and the resulting expression profiles were integrated into a GEM of human astrocytes. GEMs are stoichiometric representations of cellular metabolism that link genes, enzymes, and metabolic reactions into a unified network, enabling the simulation of metabolic fluxes under defined constraints. FBA and FVA were used to evaluate condition-specific metabolic performance and flexibility (Figure 1).

Importantly, the inferred components are sensitive to modeling assumptions, including the predefined number of cell types, the degree of transcriptional separability among cellular populations, and the transformation of microarray intensities into pseudo-counts. As a result, deconvolution may introduce noise or ambiguity, particularly for closely related or low-abundance cell types. Therefore, downstream analyses based on inferred astrocyte-associated profiles were interpreted conservatively and supported using multiple orthogonal criteria.

To ensure the robustness and interpretability of the inferred cell-type–associated transcriptional profiles, we implemented a multi-layer validation framework encompassing four complementary levels: (i) confirmation of cell-type signal presence in bulk transcriptomic data, (ii) internal validation of deconvolution stability and component reproducibility, (iii) external validation against independent single-nucleus RNA-seq datasets, and (iv) biological validation through marker enrichment and pathway-level concordance analyses. This structured approach allows distinguishing between methodological consistency, biological plausibility, and cross-platform reproducibility.

To assess the internal stability of the deconvolution results, CDSeq was executed across multiple independent runs using identical parameter settings. The resulting latent components were compared using pairwise correlation analyses to evaluate reproducibility of inferred expression profiles. Consistent identification of an astrocyte-like component across runs supported the robustness of the decomposition, although inferred profiles should be interpreted as stable approximations rather than exact reconstructions.

External validation was performed using an independent single-nucleus RNA-seq dataset of human astrocytes (Serrano-Pozo et al., 2024). Agreement was evaluated at the pathway level by comparing enriched biological processes derived from deconvolution-inferred profiles with those identified in astrocyte populations from the single-nucleus dataset. Concordance was assessed based on overlap and directional consistency of significantly enriched pathways.

To evaluate whether inferred transcriptional programs reflected astrocyte-enriched rather than global tissue-level signals, enrichment patterns were compared across all latent components identified by CDSeq. Key metabolic and stress-related pathways were predominantly enriched in the astrocyte-like component relative to other components, supporting cell-type enrichment while acknowledging the limitations of deconvolution-based inference.

### 2.1 Dataset selection and quantitative quality assessment

We compared two publicly available transcriptomic datasets from the Gene Expression Omnibus (GEO): GSE28146 (hippocampal CA1 tissue; Blalock et al., 2011) and GSE63060 [peripheral blood; (Sood et al., 2015)], both including samples from control, mild cognitive impairment (MCI), and Alzheimer’s disease (AD) subjects (Supplementary Table S1). Dataset selection was based on a combined evaluation of statistical robustness and biological relevance, particularly considering the central role of hippocampal dysfunction in early AD.

To ensure objective selection, we performed a multi-layered quantitative assessment using complementary criteria (summarized in Supplementary Table S2):

- First, we evaluated unsupervised group separability using principal component analysis (PCA) on normalized expression data. We quantified (i) the proportion of variance explained by the first two principal components and (ii) silhouette scores computed across the first k principal components (k = 2–10), using clinical labels.
- Second, we assessed supervised classification performance using linear discriminant analysis (LDA), reporting leave-one-out cross-validated accuracy and macro-averaged F1-scores to evaluate discrimination between clinical stages.
- Third, we quantified differential expression robustness by applying an identical analysis pipeline to both datasets, including log2 fold-change thresholds and false discovery rate (FDR) correction. We compared the number of differentially expressed genes (DEGs) across contrasts and evaluated the consistency of enriched biological pathways across stages.

**FIGURE 2:**
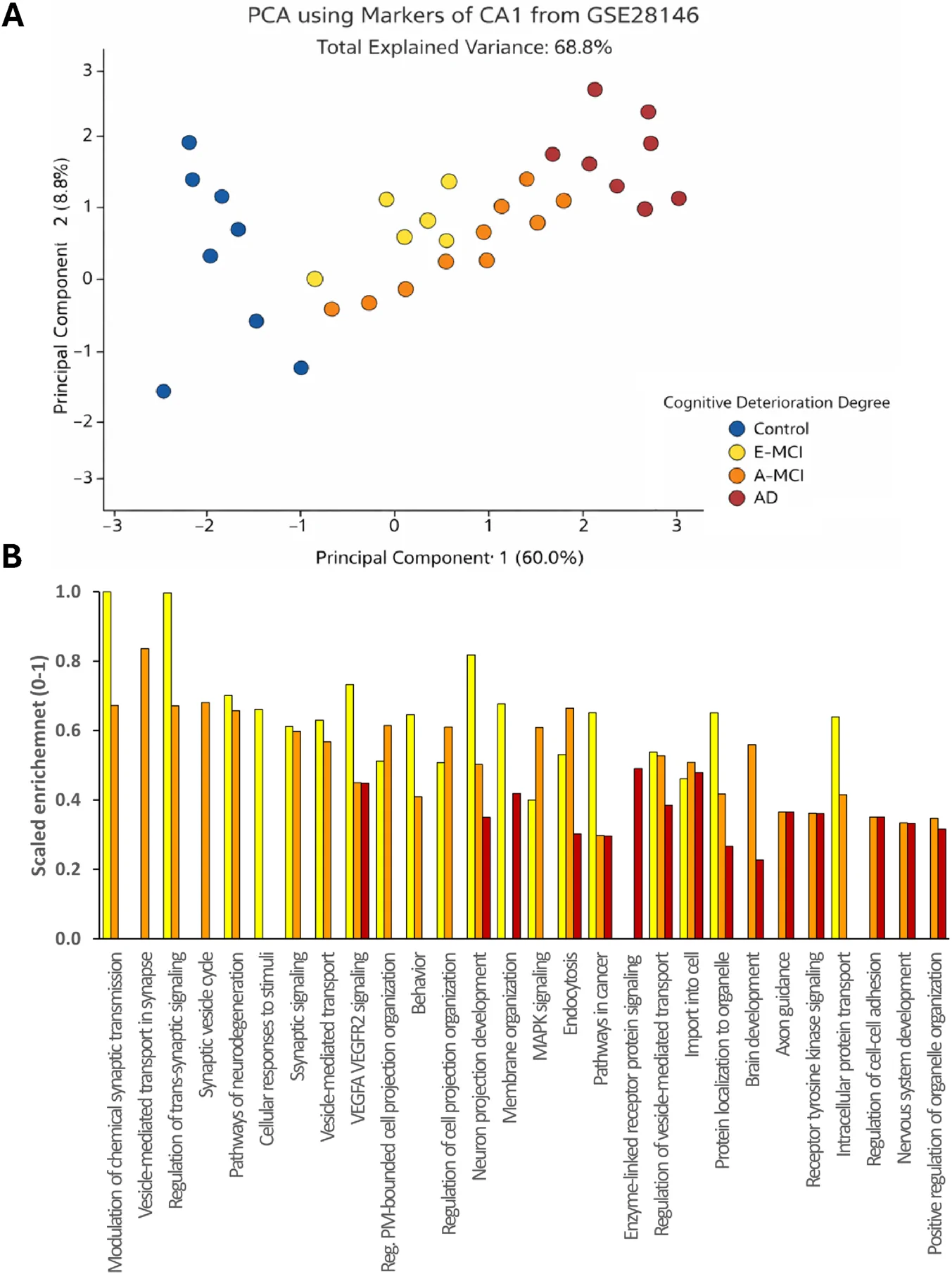
Transcriptomic progression and functional enrichment across the MCI spectrum. **(A)** Principal component analysis (PCA) of CA1 hippocampal transcriptomic markers from dataset GSE28146 after beta transformation. Samples are distributed according to clinical stage (Control, Early MCI, Advanced MCI, and AD), with a clear separation along Principal Component 1 (60% variance explained; total variance 68.8%), indicating a progressive molecular shift associated with cognitive decline. **(B)** Functional enrichment of differentially expressed genes across disease stages. Bars represent scaled enrichment scores (0–1) for Early MCI (yellow), Advanced MCI (orange), and AD (red). Enriched biological processes include synaptic transmission, vesicle-mediated transport, VEGFA–VEGFR2 signaling, neuronal projection development, and receptor-mediated signaling pathways. Early stages show stronger enrichment in signaling and synaptic regulation, whereas later stages exhibit broader but attenuated enrichment across neuronal organization and intracellular signaling pathways, consistent with progressive network-level dysfunction.

Across all evaluated criteria, GSE28146 demonstrated higher group separability, improved classification performance, and more stable transcriptional signatures compared to GSE63060 (Supplementary Figure S1). In particular, PCA revealed a progressive distribution of samples along disease stages, while LDA confirmed improved discrimination between clinical groups (Figure 2A).

In addition to quantitative metrics, biological context was considered. GSE28146 is derived from hippocampal CA1 tissue, directly relevant to early AD pathology, whereas GSE63060 originates from peripheral blood. Given that astrocyte deconvolution and metabolic modeling require tissue-specific transcriptional signals, peripheral blood data would rely on indirect exosomal or systemic signals that may not accurately reflect region-specific brain processes.

Therefore, GSE28146 was selected for downstream analyses, including PCA/LDA characterization, CDSeq-based deconvolution, and genome-scale metabolic model contextualization. Results obtained from GSE63060 are provided in the Supplementary Material for comparison.

Clinical groups in GSE28146 were defined according to the original study labels. Mini-Mental State Examination (MMSE) scores are reported as descriptive summaries rather than strict classification thresholds to contextualize disease severity:

- Healthy controls: MMSE typically ≥25
- Early MCI: MMSE approximately 20–24
- Advanced MCI: MMSE approximately 14–19
- AD: MMSE typically ≤14

### 2.2 Differential expression and functional enrichment analysis

Differential expression analysis was conducted using the GSE28146 dataset, selected for its greater sensitivity to detect subtle transcriptional changes across the Control-MCI–AD continuum (Supplementary Figure S1). Three pairwise comparisons were performed: (i) Control vs. Early MCI, (ii) Control vs. Advanced MCI, and (iii) Control vs. AD.

For each comparison, log_2_ fold change (log_2_FC) values were computed, and differential expression was assessed using a two-sided Student’s t-test followed by Benjamini–Hochberg correction to control the false discovery rate (FDR). Genes were considered differentially expressed if they met the criteria FDR <0.05 and absolute fold change >1.5 (log_2_FC > 0.585). Only genes passing these thresholds were retained for downstream analyses (Lowe et al., 2017).

The resulting sets of significantly differentially expressed genes (DEGs) were then subjected to functional enrichment analysis using the Metascape platform (Zhou et al., 2019). The background universe for enrichment consisted of all genes retained after preprocessing and filtering of the expression dataset. Enrichment analysis focused primarily on Gene Ontology Biological Process terms, and when relevant, pathway resources such as Reactome and KEGG were also considered. Default Metascape parameters were applied, including multiple-testing correction, an enrichment significance threshold of q < 0.05, a minimum overlap of three genes, and a minimum gene set size of five genes.

Enrichment results were summarized and visualized using bar plots depicting both enrichment significance and gene counts, highlighting biological processes and pathways associated with astrocyte-related transcriptional alterations along MCI progression (Figure 2B).

### 2.3 Validation of cellular profiles via correlation with reference signatures

Before applying deconvolution analysis, we validated that the bulk expression profiles from GSE28146 contained transcriptomic signatures of distinct cell types, including astrocytes. For this, we used reference expression profiles from the Allen Institute’s Human Cell Atlas, which includes aggregated gene expression data for human hippocampal cell types including astrocytes, pyramidal neurons, oligodendrocytes, microglia, and endothelial cells (https://data.humancellatlas.org/hca-bio-networks/nervous-system/atlases/brain-v1-0).

We performed the following steps:

1. Inverse transformation of microarray log_2_-intensity values to approximate pseudo-counts, mimicking relative abundance as in RNA-seq data.
2. Mapping gene identifiers from gene symbols to Ensembl IDs, retaining only overlapping genes between datasets.
3. Pearson correlation analysis between each bulk sample and the average expression profile of each reference cell type.

**FIGURE 3:**
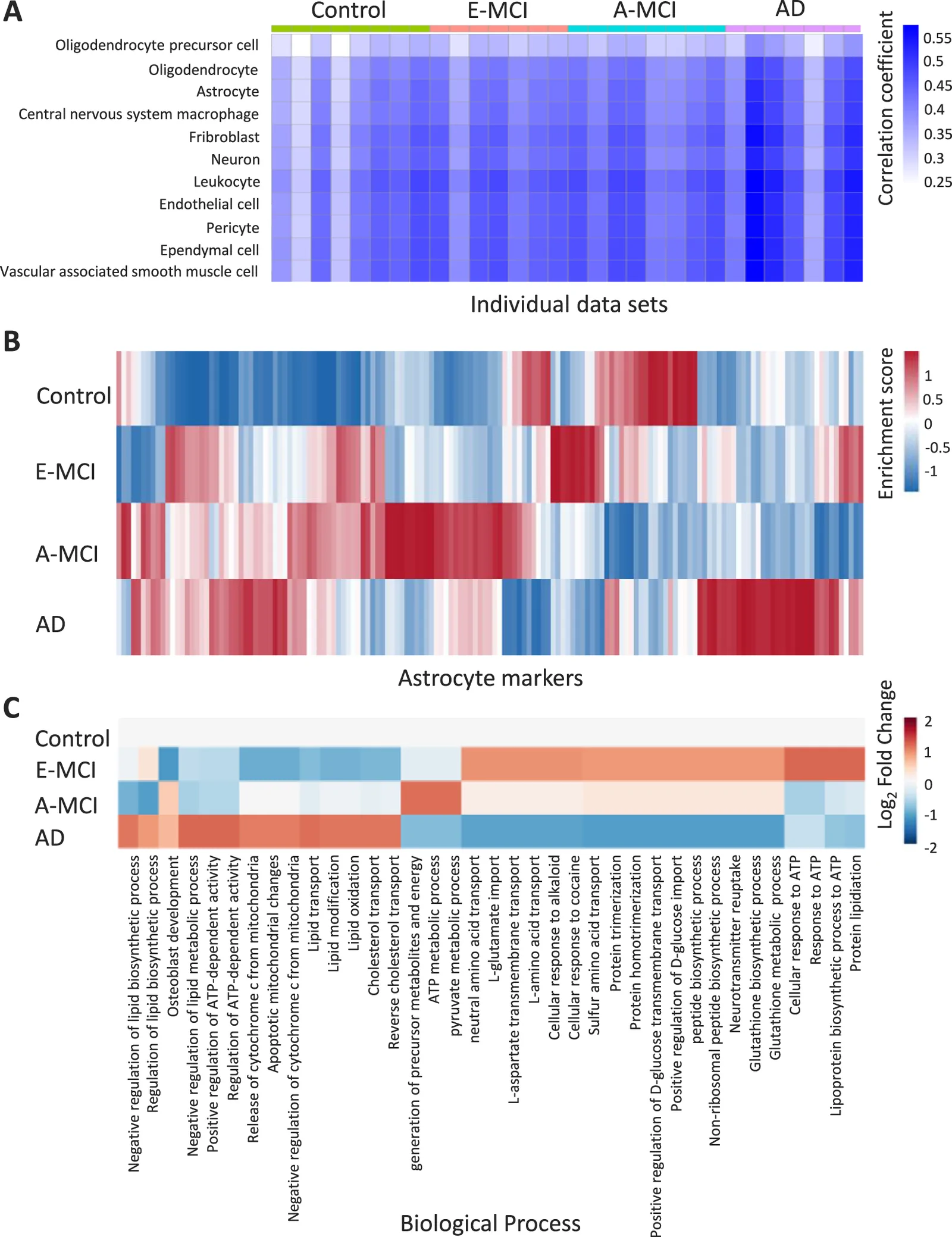
Analysis of astrocyte-specific transcriptomic profiles and functional enrichment across clinical stages. **(A)** Heatmap of correlation between transcriptomic profiles of different cell types, estimated using CDSeq deconvolution from the GSE28146 dataset. Samples are ordered by clinical condition (Control, Early MCI, Advanced MCI, and AD). Astrocyte profiles show sufficientconsistency across samples, supporting their validity for downstream modeling. **(B)** Heatmap of functionally relevant astrocytic gene expression, grouped by biological process. A progressive pattern of differential regulation is observed with increasing disease severity, particularly in genes related to cell signaling, metabolism, membrane transport, and synaptic function. **(C)** Functional enrichment analysis of differentially regulated biological processes between clinical conditions. The most representative terms are hierarchically grouped according to their behavior across Control, Early MCI, Advanced MCI, and AD conditions. Notable pathways include energy metabolism, ATP generation, mitochondrial transport, cell cycle regulation, and astrocytic signaling, especially disrupted in Advanced MCI and AD.

The resulting correlation matrix was visualized as a heatmap and complemented with boxplots to highlight cell-type-specific trends across clinical stages (Figure 3A).

This analysis revealed a strong correlation with astrocytic profiles, particularly in advanced MCI stage and AD condition. This finding supported the use of a deconvolution algorithm to extract an astrocyte-specific transcriptional profile.

### 2.4 CDSeq deconvolution and parameter optimization

We performed unsupervised deconvolution using CDSeq (Kang et al., 2021) to infer both cell-type proportions and cell-type–associated expression profiles from bulk hippocampal transcriptomic data. CDSeq models bulk gene expression as a composite signal arising from multiple latent cellular sources, enabling the simultaneous estimation of cell-type proportions and their corresponding transcriptional profiles without requiring predefined reference signatures. This unsupervised approach is particularly advantageous in the context of neurodegenerative diseases, where cell states—such as reactive astrocytes—may not be fully captured by existing reference atlases or may exhibit condition-specific transcriptional programs.

Conceptually, CDSeq decomposes the bulk expression matrix into a set of latent components, each representing a putative cell population, along with their proportional contributions across samples. Because these profiles are computationally inferred from mixed bulk signals, they should not be interpreted as direct cell-resolved measurements, but rather as model-based approximations of the underlying transcriptional contributions of distinct cellular populations (Id et al., 2019).

Importantly, the inferred components are sensitive to modeling assumptions, including the predefined number of cell types, the degree of transcriptional separability among cellular populations, and the transformation of microarray intensities into pseudo-counts. As a result, deconvolution may introduce noise or ambiguity, particularly for closely related or low-abundance cell types. Therefore, downstream analyses based on inferred astrocyte-associated profiles were interpreted conservatively and supported using multiple orthogonal criteria, including marker enrichment, component stability across runs, correlation with external reference signatures, and independent pseudobulk validation using single-nucleus RNA-seq data (Supplementary Table S3).

Our framework relies on several assumptions. First, it assumes that gene expression follows a multinomial or Dirichlet-multinomial distribution, reflecting count-based transcriptomic data. Second, it assumes that the number of cell types (K) is predefined or can be approximated, which may influence the resolution of inferred components. Third, it assumes that gene expression is sufficiently distinct across cell types to allow separation, which may not hold for closely related or low-abundance populations.

As an unsupervised method, CDSeq does not require predefined reference signatures; however, this also introduces ambiguity in component interpretation, as inferred cell types must be assigned post hoc based on marker enrichment (Supplementary Tables S4, S5). Additionally, technical noise, batch effects, and the transformation of microarray intensities into pseudo-counts may introduce uncertainty in the inferred proportions and expression profiles. Therefore, the results should be interpreted as approximations of underlying cellular composition rather than direct measurements.

Parameter optimization was conducted via a grid search aimed at minimizing reconstruction error while preserving biological interpretability and component stability. Specifically, we evaluated num_cell_types in the range of 4–10, nu between 0.01 and 0.10, and alpha between 1 and 10, while keeping total_reads fixed at 2 × 10^6^ to stabilize the pseudo-count representation of microarray intensities. For each parameter combination, CDSeq was executed across multiple independent initializations, and performance was assessed based on (i) the reconstruction error reported by the algorithm, (ii) the stability of inferred cell-type proportions across runs (Pearson and Spearman correlation), and (iii) the reproducibility of marker enrichment for known brain cell types.

Using the GSE28146 dataset, the final parameter set (num_cell_types = 6, nu = 0.05, alpha = 5, total_reads = 2 × 10^6^) provided the best balance between low reconstruction error, convergence behavior, and biologically consistent components across iterations. The algorithm inferred multiple latent components corresponding to distinct cellular populations.

The astrocytic component was identified based on the highest enrichment of well-established astrocyte markers, including GFAP, Aquaporin 4 (AQP4), and Aldehyde Dehydrogenase 1 Family Member L1 (ALDH1L1), together with additional markers listed in Supplementary Table S4 (Gorina et al., 2022; Zhang et al., 2019). Component identity was further supported by correlation with reference astrocyte expression profiles from the Allen Institute and Human Cell Atlas resources. The astrocyte-associated component was consistently recovered across all clinical conditions, including control, early MCI, advanced MCI, and AD (Figure 3B).

The resulting astrocyte-specific expression profiles were subsequently used to contextualize a genome-scale metabolic model of human astrocytes.

### 2.5 External validation of deconvolution-derived astrocyte profiles using single-nucleus RNA-seq pseudobulk analysis

To validate the biological consistency of the deconvolution results, we leveraged an independent single-nucleus RNA-seq reference dataset of astrocytes from the entorhinal cortex (EC) from controls and three stages of AD progression (Gabitto et al., 2024). EC astrocyte cells were first subsetted and aggregated into pseudobulk expression profiles using a donor-aware strategy, whereby gene expression was averaged at the individual level and subsequently across disease stages to avoid biases due to unequal cell counts. Gene expression matrices from both the deconvolution (CDSeq) and the reference dataset were log-transformed and restricted to a common set of genes.

To ensure a meaningful comparison, expression values were converted into relative changes with respect to the control condition (log-fold change) (Love et al., 2014), thereby minimizing the influence of global transcriptional programs and focusing on stage-specific biological variation. Genes with zero variance across conditions were removed, and analyses were restricted to the most variable genes to enhance sensitivity (Love et al., 2014).

Concordance between datasets was assessed using multiple complementary metrics. Pairwise Pearson correlation coefficients were computed between matched disease stages to evaluate similarity in transcriptional changes (Supplementary Figure S2). Finally, gene set enrichment analysis (GSEA) was performed for each stage relative to control to identify shared biological pathways and validate whether the deconvolution recapitulated known astrocyte responses across disease progression (Supplementary Table S4). Together, these approaches allowed us to evaluate both quantitative agreement and preservation of biological structure between deconvolved and single-cell-derived profiles.

### 2.6 Astrocyte GEM selection and curation

Astrocyte-specific gene expression profiles, obtained through CDSeq-based deconvolution of bulk microarray data (GSE28146), were integrated into a curated genome-scale metabolic reconstruction of human astrocytes (Angarita-Rodríguez et al., 2024) using a modified version of the exp2flux pipeline (Osorio et al., 2016).

Because the original data corresponds to microarray-derived expression intensities rather than RNA-seq counts, expression values were first normalized and transformed into pseudo-counts to approximate relative transcript abundances. These values were subsequently used as proxies for gene expression and are hereafter referred to as TPM-like units for modeling purposes. Importantly, these values do not represent absolute transcript quantification but rather relative expression levels suitable for constraining metabolic models.

Deconvoluted astrocyte-specific expression profiles were mapped to metabolic reactions using Gene–Protein–Reaction (GPR) rules (Filippo et al., 2025). Gene activity was defined using a threshold equivalent to TPM >1.0, ensuring consistency with constraint-based modeling approaches while accounting for the relative nature of the expression estimates. Logical relationships encoded in GPR rules were resolved such that reactions associated with OR-linked genes (e.g., isozymes) were considered active if at least one gene was expressed, whereas reactions associated with AND-linked genes (e.g., protein complexes) required all corresponding genes to be active.

Expression-derived constraints were incorporated into the metabolic network by restricting flux bounds according to gene activity. Reactions associated with inactive genes were constrained to zero flux, while reactions linked to active genes were assigned upper bounds proportional to their relative expression levels, following an E-Flux–like strategy (Kim et al., 2016).

The resulting condition-specific models were analyzed within the framework of genome-scale metabolic modeling (GEM), where metabolism is represented as a system of stoichiometric reactions under mass-balance constraints (Di et al., 2022; Qi et al., 2014). Assuming a steady-state condition, feasible flux distributions were computed using flux balance analysis (FBA) (Orth et al., 2010). This approach enables the translation of transcriptomic variation into condition-specific metabolic phenotypes, facilitating the comparison of metabolic states across disease stages. It is important to note that these models predict feasible flux distributions rather than intracellular metabolite concentrations.

This procedure yielded four contextualized metabolic models representing astrocytic metabolic states in Control, Early MCI, Advanced MCI, and Alzheimer’s disease (AD) conditions.

### 2.7 Flux balance analysis and flux variability analysis

Each model was analyzed using FBA to estimate steady-state flux distributions under condition-specific constraints. The objective function was astrocyte biomass synthesis, used here as a proxy for integrated metabolic maintenance requirements rather than cellular proliferation. This formulation captures the coordinated production of cellular components essential for astrocyte homeostasis and neuronal support (Table 2), including metabolites involved in neurotransmitter cycling, membrane integrity, redox balance, and energy metabolism (Orth et al., 2010).

**TABLE 2:** Simulated metabolic fluxes in astrocyte models across clinical conditions. Metabolic flux values (in mM gDW^−1^ h^−1^) estimated from growth simulations in transcriptome-contextualized astrocyte models under Control, Early MCI, Advanced MCI, and AD clinical scenarios. A progressive reduction in the biomass-derived maintenance proxy is predicted up to Advanced MCI, with a slight increase in the AD stage.

Scenario	Metabolic fluxes (mMgWD^−1^ h^-1^)
Experimental*	0.32
Control	0.3304
Early MCI	0.3211
Advanced MCI	0.2718
AD	0.3037

* *in vitro* growth flux of 0.32 mM gDW−1 h−1 reported by (Prah et al., 2019).

FVA was used to compute the allowable flux ranges for each reaction under the same constraints. This enabled the assessment of metabolic flexibility and identification of alternative pathways potentially activated under stress conditions (Angarita-Rodríguez et al., 2024).

It is important to note that flux balance analysis generates deterministic solutions based on constraint optimization and does not inherently provide statistical measures of variability or significance. Therefore, differences between conditions were interpreted based on relative changes in flux distributions and consistency across complementary analyses rather than formal statistical testing.

To further assess the biological relevance of the predicted metabolic flux alterations, a targeted comparison was performed between GEM-derived flux changes and experimental observations from APOE genotype–dependent astrocyte metabolic profiling (Lysaker et al., 2025). This study employed stable isotope tracing and bioenergetic assays in human iPSC-derived astrocytes, providing an experimental framework to evaluate central carbon metabolism, redox balance, and mitochondrial function.

For this comparison, only reactions with direct biological correspondence to experimentally measured metabolites or pathways were retained. These included reactions associated with glutamate metabolism, tricarboxylic acid (TCA) cycle activity, glycolysis, and redox-related processes.

Flux changes in the GEM were normalized relative to the control condition (APOE3-like state) using the following formulation (Equation 1):

$$Relative\;change\;\left(\%\right)=\frac{|Fluxcondition-Fluxcontrol}{|Fluxcontrol|}\;x\;100$$ (1)

This normalization enabled a quantitative comparison of the magnitude and direction of metabolic alterations between the model-derived predictions and experimental observations under APOE4-like (incipient) conditions. The resulting comparisons are summarized in Table 5.

**TABLE 3:** Reaction-level flux alterations in astrocyte-specific metabolic models across the MCI–AD continuum and their association with reactive astrocyte-related biological processes. Selected reactions showing flux changes across Control, Early MCI, Advanced MCI, and AD conditions are presented. Flux values correspond to model-predicted steady-state reaction rates derived from expression-constrained genome-scale metabolic models. Each reaction is linked to biological axes previously associated with astrocyte reactivity, including mitochondrial stress, glycolytic rewiring, lipid metabolism, nucleotide turnover, and inflammatory pathways. Interpretations are supported by literature evidence, highlighting the consistency between predicted metabolic alterations and known features of reactive astrocyte states.

Reaction Id	Reaction name	Subsystem	Control	Early MCI	Advanced MCI	AD	Associated reactive/inflammatory axis	Interpretation	References
MALtm	Malate transport (mitochondrial)	Mitochondrial transport	−10.83	9.25	7.02	2.16	Mitochondrial stress/ROS (GFAP, VIM axis)	Consistent with mitochondrial rewiring and altered redox balance, consistent with reactive astrocyte states	PMID: 32105849
ENO	Enolase	Glycolysis	−9.80	0.78	0.64	0.72	Glycolytic shift (LDHA axis)	May suggest metabolic reprogramming toward altered glycolysis, commonly observed in reactive astrocytes	PMID: 40145993
PGM	Phosphoglycerate mutase	Glycolysis	9.80	−0.78	−0.64	−0.72	Glycolytic regulation	Could reflect disruption of glycolytic homeostasis across disease stages	PMID: 40145993
CYTK6	Cytidylate kinase	Nucleotide metabolism	−9.82	3.67	3.43	3.30	Cellular stress/transcriptional activation (STAT3-related)	Compatible with increased nucleotide turnover linked to cellular stress responses	PMID: 25673868
FACOAL1832	Fatty-acid CoA ligase	Fatty acid oxidation	−9.34	−0.77	0.01	−0.71	Lipid metabolism (APOE/ABCA1 axis)	May suggest altered lipid utilization, consistent with astrocyte reactivity and lipid dysregulation	https://doi.org/10.1016/j.celrep.2020.108572
FACOAL205	Fatty-acid CoA ligase	Fatty acid oxidation	−9.28	0.78	−0.64	−0.72	Lipid remodeling	Consistent with dynamic lipid metabolic rewiring during disease progression	https://doi.org/10.1016/j.celrep.2020.108572
UTPtn	UTP nuclear transport	Nuclear transport	−9.21	−0.78	−0.63	0.72	Transcriptional reprogramming (NF-κB/STAT3 indirect)	May suggest altered nucleotide transport associated with activation states	PMID: 25673868
r1666	Y + LAT2 transport	Amino acid transport	−2.12	189,733.00	8.34	−1.39	Amino acid metabolism (SLC transporters)	Strong early-stage alteration may suggest adaptive metabolic response	https://doi.org/10.3389/fphar.2022.1042989
r1637	Amino acid–polyamine transporter	Polyamine metabolism	−1.48	−19.58	−9.28	−2.45	Polyamine–inflammation axis (ODC1, SAT1)	Supports polyamine dysregulation is associated with neuroinflammatory states	PMID: 40210470
HMR_2982	Long-chain fatty acid CoA ligase	Lipid synthesis	9.73	0.78	0.64	0.72	Lipid synthesis (APOE-related)	Supports disruption of astrocytic lipid homeostasis in disease	https://doi.org/10.1016/j.celrep.2020.108572

It is important to note that flux balance analysis generates deterministic solutions based on constraint optimization and does not inherently provide statistical measures of variability. Therefore, differences between conditions were interpreted as consistent model-supported trends rather than statistically inferred differences.

To evaluate the biological relevance of model-derived metabolic predictions, we implemented a structured validation strategy integrating independent experimental data from isotope-resolved metabolomics in human iPSC-derived astrocytes. Validation was performed at three levels: (i) reaction-level directional agreement, (ii) pathway-level consistency across major metabolic modules, and (iii) system-level convergence with known metabolic phenotypes.

Concordance was defined based on the directionality and qualitative behavior of metabolic changes. Reactions were considered concordant when predicted flux changes aligned with experimental trends, and partially concordant when agreement was observed at the pathway level without direct one-to-one correspondence.

Validation analyses were restricted to pathways with direct experimental correspondence, which may introduce selection bias. Therefore, agreement should be interpreted as pathway-specific rather than global validation of the metabolic network.

## 3 Results

### 3.1 Transcriptomic differentiation and functional enrichment across disease stages

PCA of transcriptomic profiles from the CA1 hippocampal region (GSE28146) revealed a clear separation among clinical conditions (Figure 2A). Control and AD samples occupied opposing regions of the projection space, while Early and Advanced MCI samples were distributed in intermediate positions. This stepwise organization reflects a gradual transcriptomic shift across disease stages and supports the biological relevance of MCI as a transitional state rather than a discrete category.

Functional enrichment analysis of differentially expressed genes identified stage-dependent alterations in biological processes (Figure 2B). Early MCI was characterized by enrichment of enzyme-linked receptor signaling pathways, including VEGFA–VEGFR2 signaling, as well as processes related to membrane organization and axon guidance. Advanced MCI exhibited a shift toward pathways associated with synaptic transmission, trans-synaptic signaling, and vesicle-mediated communication. In AD, enrichment patterns reflected widespread disruption of synaptic vesicle cycling, endocytosis, Mitogen-Activated Protein Kinase (MAPK) signaling, oxidative stress responses, and neuroinflammatory pathways. Together, these results delineate a progressive molecular trajectory from early signaling dysregulation to synaptic failure and inflammatory activation as cognitive impairment advances.

### 3.2 Astrocyte-specific transcriptomic remodeling

Unsupervised deconvolution of bulk hippocampal transcriptomes using CDSeq identified six latent cellular components, corresponding to astrocytes, neurons, oligodendrocyte-like cells, microglia-like cells, endothelial-like cells, and one mixed glial population (Figure 3A). The number of inferred components is lower than the number of reference cell types examined in the correlation analysis, reflecting a known limitation of unsupervised deconvolution methods applied to heterogeneous bulk brain tissue, where transcriptionally similar or low-abundance populations may not be reliably separated.

The astrocyte-associated component was inferred based on the enrichment of canonical astrocyte markers, including Glial Fibrillary Acidic Protein (GFAP), AQP4, ALDH1L1, and additional markers listed in Supplementary Table S3. Absolute normalized expression values for these markers are reported explicitly in Supplementary Table S3 for each clinical condition, confirming their consistent expression across stages and supporting the biological validity of the extracted astrocytic signatures as inputs for metabolic modeling.

The enrichment scores displayed in Figure 3B represent relative marker enrichment within each inferred CDSeq component and are intended to support component identity rather than stage-wise differential expression. Stage-dependent transcriptional changes were assessed separately through differential expression and functional enrichment analyses (Figure 3C).

CDSeq was applied jointly to all samples within each clinical condition, yielding a single representative astrocytic transcriptomic profile per condition. Individual samples were not deconvolved separately. This strategy reduces noise and improves component stability when analyzing bulk microarray data.

Because these profiles were inferred from bulk transcriptomic mixtures rather than measured directly at single-cell resolution, the resulting astrocyte-associated signatures should be interpreted as latent approximations of cell-type–enriched expression programs. This limitation is particularly relevant for subtle stage-dependent differences, which may be influenced by inference noise and partial overlap with other glial populations.

Astrocytic transcriptomes exhibited stage-dependent functional remodeling (Figure 3C). Control astrocytes were enriched for processes supporting synaptic maintenance, energy metabolism, vesicle-mediated signaling, and antioxidant defense. In Early and Advanced MCI, astrocytic profiles shifted toward transcriptional programs associated with inflammation, cytoskeletal remodeling, and stress responses. In AD, genes involved in neurotransmitter cycling and redox homeostasis were markedly downregulated, consistent with reduced expression of programs associated with astrocytic homeostatic and neuroprotective functions.

### 3.3 External validation of astrocyte-specific deconvolution

To evaluate the biological plausibility of the astrocyte-specific signatures inferred by CDSeq, we performed an external validation using single-nucleus RNA-seq data from the entorhinal cortex (EC) astrocyte dataset reported by Serrano et al. Astrocyte profiles were aggregated into stage-specific pseudobulks and compared across disease progression.

Because the external EC astrocyte dataset used a different staging framework than the MMSE-based classification in GSE28146, comparisons were interpreted at the level of trajectory and functional concordance rather than direct one-to-one stage equivalence. Nevertheless, Stage 1 included healthy samples, while Stage 4 corresponded to AD patients.

As shown in Supplementary Figure 2A, global transcriptomic patterns derived from CDSeq-based astrocyte profiles exhibit a structured organization across stages, with genes displaying coordinated shifts in expression that reflect stage-dependent changes in astrocyte-associated transcriptional programs.

To quantitatively assess concordance, Pearson correlations were computed between stage-specific astrocyte expression profiles derived from CDSeq and pseudobulk astrocyte profiles from single-nucleus RNA-seq data, using shared genes and relative expression changes with respect to control. As shown in Supplementary Figure 2B, the dominant pattern corresponds to diagonal associations between equivalent stages: control with Stage 1, early MCI with Stage 2, advanced MCI with Stage 3, and AD with Stage 4. Correlations across non-matching stages were generally lower, reflecting differences in transcriptional states across disease progression.

Functional enrichment analysis further supported these observations (Supplementary Figure 2C). Although complete pathway overlap between datasets was not observed, consistent stage-associated functional themes emerged. Early stages were enriched in extracellular organization, hypoxia response, and early stress adaptation pathways; intermediate stages showed enrichment in proteostasis and protein folding; and late stages were characterized by signaling, hormone-related processes, and calcium-mediated pathways.

Importantly, although gene-level concordance between datasets was moderate, the recurrence of shared functional modules—particularly those related to proteostasis and stress responses—supports agreement at the pathway level.

### 3.4 Context-specific astrocyte metabolic models

Astrocyte-specific transcriptomes were integrated into a curated genome-scale metabolic model of human astrocytes to generate four condition-specific models representing Control, Early MCI, Advanced MCI, and AD states. The resulting models encompassed 6,520 metabolites and 10,586 reactions and were constrained using an expression-informed strategy analogous to the E-Flux framework.

Reaction bounds were scaled proportionally to transcript abundance, while absolute flux units (mmol·gDW^-1^·h^-1^) emerged from the interaction between expression-scaled constraints, reaction stoichiometry, exchange constraints, and the objective function during optimization. GPR rules were handled explicitly: for OR relationships, the maximum expression value among associated genes was used; for AND relationships, the minimum expression value was applied. Reactions without associated genes were left unconstrained. A minimum expression threshold of TPM ≥1 was used to define gene activity, consistent with prior studies of transcriptional activity in bulk datasets.

**FIGURE 4:**
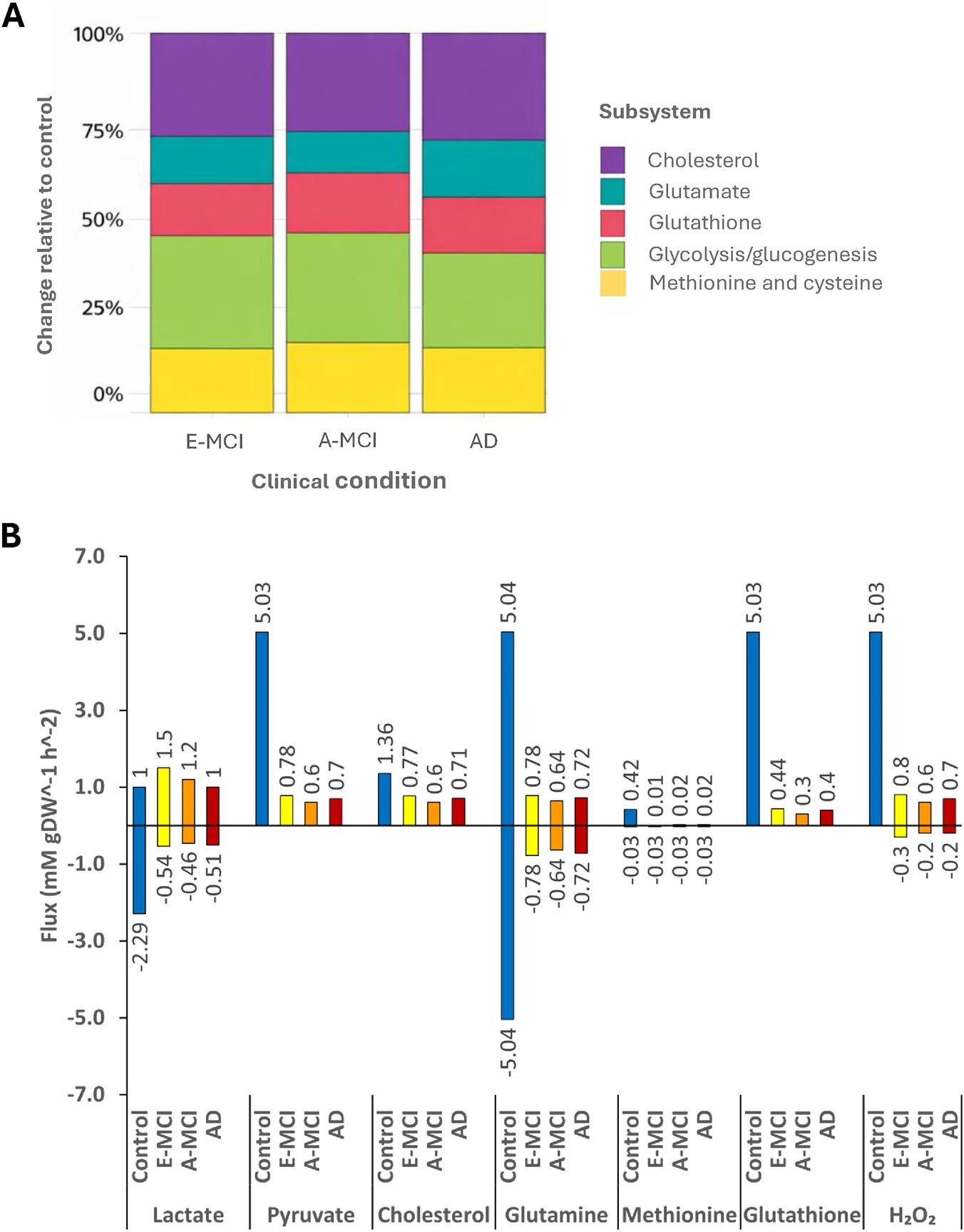
Metabolic flux changes associated with the progress of MCI to AD. Panel **(A)** shows the percentage of reactions that exhibit flux changes compared to the control group, categorized by metabolic subsystems—specifically cholesterol metabolism, glutamate metabolism, glutathione metabolism, glycolysis/gluconeogenesis, and methionine and cysteine metabolism—across Early and Advanced MCI to AD. FVA results **(B)** display key metabolites under each clinical condition relative to the control: lactate, pyruvate, cholesterol, glutamine, methionine, glutathione, and hydrogen peroxide (H_2_O_2_). In these panels, blue and red dots represent the minimum and maximum flux bounds (mmol gDW^−1^ h^−1^), with numeric values indicating the allowed flux range for each condition. This analysis is consistent with a progressive reduction in metabolic flexibility and subsystem-specific alterations as MCI advances to AD.

FBA predicted a progressive reduction in the biomass/maintenance objective from Control to Advanced MCI, followed by a partial increase in AD (Table 4). Biomass flux was interpreted as a proxy for metabolic maintenance capacity rather than cellular proliferation. FVA, performed without constraining solutions to maximal biomass production, revealed a progressive narrowing of allowable flux ranges across disease stages, indicating reduced metabolic flexibility (Figure 4B).

**TABLE 4:** Selected metabolic reactions showing marked alterations in flux values (in mM gDW^-1^ h^-1^) across the progression of MCI to AD. These reactions belong to key biochemical pathways, including folate metabolism, glutamate and glutathione turnover, glycolysis, pyruvate metabolism, and cholesterol biosynthesis. The differential flux patterns observed suggest the presence of metabolic inflection points and regulatory bottlenecks that may underlie astrocyte–neuron metabolic dysregulation and contribute to the pathophysiology of MCI and AD. This selection emphasizes reactions with consistent directional trends or abrupt shifts indicative of stage-specific metabolic reprogramming.

Reaction	Subsystem	Control	Early MCI	Advanced MCI	AD
Putrescine reductase	Methionine and cysteine metabolism	−3.60	0.29	0.0	0.0
(Putrescine-Forming)	Arginine and proline metabolism	3.608	−1,34E-02	0.0	0.0
Exchange of L-Arginine	Exchange/demand reaction	1.50	0.77	0	−0.723
Methylenetetrahydrofolate dehydrogenase (NADP)	Folate metabolism	−5.03	0.77	0.637	0.72
Methylenetetrahydrofolate dehydrogenase (NAD)	Folate metabolism	5.03	−0.77	−0.637	−0.72
5,10-Methylenetetrahydrofolatereductase (NADPH)	Folate metabolism	3,85	0.0	0.0	0.0
5-Methyltetrahydrofolate:NAD + Oxidoreductase	Folate metabolism	3,64	−0.13	0.0	0.006
Glutaminase	Glutamate metabolism	5.03	0.77	0.63	0.72
Glutamate dehydrogenase (NAD) mitochondrial	Glutamate metabolism	5.03	−0.77	0.63	0.64
Glutamate dehydrogenase (NADP) mitochondrial	Glutamate metabolism	−5.03	0.69	−0.63	−0.72
Succinate-Semialdehyde:NAD + Oxidoreductase	Glutamate metabolism	−2.78	−1.19	−0.89	−0.72
Glutathione:NAD + Oxidoreductase	Glutathione metabolism	2.76	0.73	0.63	−0.29
Gamma-Glutamyltranspeptidase	Glutathione metabolism	1.61	0.77	0.63	0.72
L-lactate dehydrogenase	Pyruvate metabolism	5.03	0.10	−0.63	−0.63
Triose-Phosphate Isomerase	Glycolysis/gluconeogenesis	7.38	−0.43	−0.43	−0.19
(S)-Lactate:NAD + Oxidoreductase	Glycolysis/gluconeogenesis	4.81	0.58	0.21	−0.32
Acetyl Coenzyme A C-Acetyltransferase	Cholesterol metabolism	7.91	0.77	0.63	0.72
24-Dehydrocholesterol Reductase [Precursor]	Cholesterol metabolism	9.85	0.0	0.004	0.72
Sterol 14Alpha-Demethylase	Cholesterol metabolism	0.01	−0.01	−0.008	0.02
Methylsterol Monooxygenase	Cholesterol metabolism	0.01	−0.01	−0.008	0.02
3Beta-Hydroxysteroid 3-dehydrogenase	Cholesterol metabolism	0.01	−0.01	−0.008	0.02
Cholestenol Delta-Isomerase	Cholesterol metabolism	0.01	−0.01	−0.008	0.02
Cholesterol Monooxygenase	Cholesterol metabolism	−0.55	−0.77	−0.63	−0.72
Delta24-Sterol Reductase	Cholesterol metabolism	−9.85	0.0	−0.004	−0.72

**TABLE 5:** Comparison of genome-scale model (GEM)-derived flux changes with experimental metabolic alterations observed in APOE4 iPSC-derived astrocytes (Lysaker et al., 2025). Fluxes were normalized to the control (APOE3-like) condition and expressed as percentage change relative to control. Only reactions with direct biological correspondence to experimentally measured pathways (glutamate metabolism, TCA cycle, and glycolysis) were included. Concordance indicates qualitative agreement between GEM predictions and experimental observations, while non-assessable denotes pathways not represented in the model subset.

Metabolic module	Experimental evidence (APOE4 astrocytes)	GEM reaction proxy	Control flux	APOE4-like flux	Flux direction change	Δ% vs. control	Biological interpretation	Concordance
Glutamate metabolism	Increased glutamate labeling and altered carbon routing	Glutaminase	5.03	0.77	No	−84.7%	Strong reduction in glutamate production flux, indicating impaired glutamate metabolism	Partial
Glutamate mitochondrial redox	Mitochondrial dysfunction and altered glutamate oxidation	Glutamate dehydrogenase (NAD)	5.03	−0.77	Yes (reversal)	−115.3%	Flux reversal suggests reorganization of mitochondrial glutamate metabolism	Partial
Glutamate redox balance	Altered redox coupling	Glutamate dehydrogenase (NADP)	−5.03	0.69	Yes (reversal)	+113.7%	Opposite-direction flux indicates major redox rewiring	Partial
TCA cycle	Reduced carbon incorporation into succinate	Succinate-semialdehyde dehydrogenase	−2.78	−1.19	No	+57.2%	Decreased absolute flux consistent with reduced TCA throughput	Yes

Subsystem-level analysis showed that reactions related to glutamate–glutamine cycling, glutathione metabolism, glycolysis/gluconeogenesis, cholesterol metabolism, and one-carbon metabolism exhibited the most pronounced reductions in flux variability (Supplementary Table S6).

These changes are further supported by the distribution of active reactions across metabolic subsystems, which revealed a consistent dominance of extracellular transport processes alongside stage-dependent shifts in lipid metabolism, mitochondrial transport, and fatty acid oxidation pathways (Supplementary Figure S3).

It is important to note that constraint-based models do not directly predict intracellular metabolite concentrations. Accordingly, references to glutamate, glutamine, or other metabolites in this study reflect changes in reaction fluxes or exchange capacities, which may influence—but do not directly equate to—steady-state intracellular concentrations.

### 3.5 Comparative validation of GEM-derived flux changes against APOE-dependent astrocyte metabolic profiling

To further evaluate the biological relevance of the predicted metabolic alterations, we compared GEM-derived flux changes with experimental observations from APOE4 astrocytes obtained through isotope-resolved metabolomics (Lysaker et al., 2025).

Within the glutamate metabolic module, the model predicted a strong reduction in glutaminase flux, with an 84.7% decrease relative to the control condition. This result indicates a reduced conversion of glutamine to glutamate within the modeled system. In parallel, experimental data from APOE4 astrocytes showed increased labeling of glutamic acid isotopologues, reflecting altered carbon routing through TCA-related pathways.

At the level of mitochondrial glutamate metabolism, the model predicted an inversion of flux direction in NAD-dependent glutamate dehydrogenase. Similarly, the NADP-dependent glutamate dehydrogenase reaction also exhibited inversion of flux direction, indicating changes in reactions associated with redox metabolism.

In contrast, TCA cycle-associated reactions showed a reduction in flux magnitude, particularly in succinate-related metabolism. This pattern is consistent with reduced incorporation of glucose-derived carbon into TCA intermediates as observed experimentally in APOE4 astrocytes.

Together, these results indicate partial concordance between model-predicted flux alterations and experimentally observed metabolic changes in APOE4 astrocytes, particularly in pathways related to glutamate metabolism, mitochondrial function, and central carbon metabolism.

## 4 Discussion

### 4.1 Transcriptomic progression and molecular trajectories across the MCI–AD

PCA applied to transcriptomic data from the GSE28146 dataset for the CA1 region of the hippocampus revealed a clear separation between the clinical conditions (Figure 2B). Control and AD samples occupied opposite quadrants, while Early and Advanced MCI samples were distributed in intermediate positions, consistent with a gradual reorganization of gene expression along the disease continuum. This spatial arrangement supports the notion that MCI represents a biologically meaningful transitional stage rather than a discrete or homogeneous condition.

Importantly, this transcriptomic progression provides a rationale for integrative approaches that move beyond differential expression alone. While PCA captures global variance, it does not directly inform on functional consequences. Therefore, combining enrichment analyses with astrocyte-specific metabolic modeling offers a complementary strategy to interpret how gradual transcriptional shifts may translate into altered cellular functionality.

### 4.2 Stage-dependent disruption of molecular pathways in MCI and AD

Functional enrichment analyses comparing Early MCI, Advanced MCI, and AD samples to controls (Figure 2B) revealed a sequential and cumulative disruption of biological processes across disease stages. Early MCI was primarily associated with altered enzyme-linked receptor signaling, particularly Receptor Tyrosine Kinase (RTK) pathways, including VEGFA–VEGFR2 signaling. These pathways regulate synaptic plasticity, neuronal survival, and neurovascular coupling (Dickerson et al., 2005; Morris and Cummings, 2005) and are frequently interpreted as early adaptive or compensatory responses (Bardy et al., 2015; Dityatev et al., 2010).

The enrichment of VEGFA–VEGFR2 signaling in Early MCI is consistent with transcriptomic studies reporting VEGFA upregulation in prodromal cognitive impairment (Figure 2B) (Luck et al., 2019; Mahoney et al., 2021). Such changes may reflect early attempts to maintain cerebral perfusion and synaptic integrity under emerging metabolic stress. Additional enrichment of membrane organization and axon guidance processes suggests early alterations in cellular architecture and signal propagation, potentially contributing to synaptic instability.

In Advanced MCI (Figure 2C), the enrichment profile shifted toward pathways directly involved in synaptic transmission and structural plasticity, including modulation of chemical synaptic signaling, vesicular communication, and trans-synaptic signaling. These findings align with reports of impaired glutamatergic and GABAergic neurotransmission in advanced MCI (Angelucci et al., 2010; Liu et al., 2019). Notably, reduced expression of Soluble N-ethylmaleimide-Sensitive Factor Attachment Protein Receptor (SNARE) complex components (e.g., VAMP1, STX1A, SNAP25) has been consistently associated with synaptic dysfunction and was also observed in transcriptomic studies of progressive cognitive impairment (Canchi et al., 2019; Jęśko et al., 2021).

In AD (Figure 2D), enrichment analyses indicated a collapse of synaptic and intracellular trafficking machinery, with overrepresentation of pathways related to vesicle-mediated transport, endocytosis, and regulation of synapse structure. These alterations parallel proteomic observations of synaptic protein loss, including reduced synaptophysin and neurofilament light chain levels (Jęśko et al., 2021), and are consistent with widespread oxidative stress, tau pathology, and mitochondrial dysfunction reported in late-stage disease (Danysz and Parsons, 2012; Toral-Rios et al., 2020).

Together, the enrichment trajectories shown in Figure 2B describe a progressive molecular disintegration that begins with signaling dysregulation, advances through synaptic remodeling and neurotransmission deficits, and culminates in profound synaptic and structural breakdown.

### 4.3 VEGFA–VEGFR2 signaling as a persistent neurovascular axis

Across all disease stages, VEGFA–VEGFR2 signaling emerged as a consistently enriched pathway (Figure 2B). Beyond angiogenesis, VEGFA plays critical roles in synaptic modulation and astrocyte–endothelial communication. The elevated representation of this pathway in Early and Advanced MCI may indicate a compensatory neurovascular response aimed at counteracting hypoxia or metabolic insufficiency (Mahoney et al., 2021; Wang J. et al., 2020; Wang X. et al., 2020).

Pharmacological studies support the functional relevance of this axis; for example, acetylsalicylic acid has been shown to downregulate VEGFR2 and modulate pathological angiogenesis (Abhinand et al., 2016). While the role of VEGFA signaling in MCI remains incompletely understood, it is unlikely to be a passive process; its persistence into AD suggests a transition from adaptive to maladaptive neurovascular remodeling. This biphasic behavior mirrors observations from transcriptomic and imaging studies and highlights VEGFA signaling as a potential stage-dependent therapeutic target (Guan et al., 2023; Zhu et al., 2021).

### 4.4 Astrocyte-specific transcriptomic reprogramming across disease stages

Unsupervised deconvolution of bulk transcriptomic data using CDSeq enabled the extraction of cell-type–specific expression components. A distinct astrocytic signature was identified based on enrichment of canonical markers such as GFAP, ALDH1L1, and AQP4, and validated through correlation with reference datasets from the Allen Institute for Brain Science (Figure 3A; Supplementary Table S4). This astrocytic component was consistently recovered across all clinical conditions.

Stratification of astrocytic transcriptomes by clinical stage (Figure 3B) revealed a progressive remodeling of astrocyte-associated functions. In control samples, astrocytes exhibited transcriptional programs consistent with homeostatic roles, including synaptic support, energy metabolism, membrane transport, and antioxidant defense. This interpretation is further supported by recent single-nucleus transcriptomic studies, which have reported increased abundance of astrocyte subtypes, upregulation of GFAP, and activation of signaling pathways associated with reactive astrocyte states across AD progression (Brugulat-serrat et al., 2023; Jurga et al., 2021).

Enrichment analysis of differentially expressed astrocytic genes (Figure 3C) further supported this transition. Processes related to glutamate clearance, ATP production, and vesicle-mediated signaling were enriched in controls but progressively diminished with disease severity. Conversely, pathways associated with oxidative stress responses and loss of synaptic maintenance became increasingly prominent in MCI and AD, consistent with the emergence of dysfunctional or reactive astrocyte phenotypes (Calì et al., 2024; Darmanis et al., 2015).

### 4.5 Interpretation and limitations of the pseudobulk validation

The external validation using the astrocyte dataset from (Serrano-Pozo et al., 2024) provides important insights into the biological interpretability of the astrocyte-specific signals inferred through CDSeq. While a direct one-to-one correspondence between bulk deconvolution and single-cell profiles is inherently limited, we observed a convergence at the pathway level across datasets.

Specifically, key biological processes—rather than individual genes—showed consistent enrichment patterns, supporting the robustness of the inferred astrocyte-associated programs. This type of functional-level agreement has been previously described as a reliable indicator of biological consistency across transcriptomic platforms, particularly when comparing bulk and single-cell data (Kang et al., 2021).

Rather than demonstrating a strict transcriptional concordance, the results highlight a functional-level agreement, where key biological processes - such as proteostasis, metabolic adaptation, and stress response - are consistently represented across both approaches. This is particularly relevant given the well-known gap between transcriptomic resolution and functional cellular states, where similar biological outcomes may arise from distinct gene-level configurations (Liu et al., 2016; Vogel and Marcotte, 2012).

The stage-dependent patterns observed suggest progressive reorganization of astrocyte function during disease progression. Early stages appear dominated by adaptive responses, including extracellular remodeling and hypoxia-related processes, potentially reflecting compensatory mechanisms. In contrast, intermediate stages show a marked activation of protein folding and quality control pathways, consistent with increased proteostatic burden. Late stages exhibit broader dysregulation, including signaling and secretory processes, which may reflect systemic alterations in astrocyte–neuron communication (Escartin et al., 2021; Liddelow et al., 2017).

Importantly, these findings should not be interpreted as definitive evidence of discrete astrocyte state transitions. Instead, they support a model of gradual functional reconfiguration, where astrocytes shift along a continuum of states. This interpretation aligns with recent single-cell studies showing that astrocyte heterogeneity is better described as a spectrum of reactive and homeostatic features rather than binary states (Escartin et al., 2021; Habib et al., 2020).

Importantly, several limitations should be acknowledged. First, CDSeq infers cell-type-specific signals under model assumptions that may introduce noise or bias. Second, differences in brain regions, disease staging criteria, and data modalities (bulk vs. single-nucleus) limit direct comparability. Third, the enrichment-based validation approach emphasizes functional convergence rather than gene-level agreement, which may obscure finer transcriptional discrepancies (Newman et al., 2019).

Despite these limitations, the integration of deconvolution and single-cell data provides a complementary framework to investigate astrocyte biology in neurodegeneration. By bridging bulk and single-cell perspectives, this approach enables the identification of robust, functionally coherent signatures that may not be apparent when each dataset is analyzed in isolation (Abdelaal et al., 2019; Keren-Shaul et al., 2017).

### 4.6 Metabolic consequences of astrocytic transcriptomic alterations

The integration of astrocyte-specific transcriptomic profiles into genome-scale metabolic models provides a functional framework to interpret how stage-dependent gene expression changes may translate into metabolic alterations across the MCI–AD continuum. Rather than representing direct biochemical measurements, these models define feasible metabolic states constrained by transcriptional activity, enabling the exploration of condition-specific metabolic reprogramming.

A central observation of this study is the progressive reduction in biomass-associated flux from Control to Advanced MCI (Table 2), suggesting impaired astrocytic metabolic maintenance capacity. The partial increase observed in AD is unlikely to reflect restored physiological function. Instead, it may represent a model-predicted reconfiguration of metabolic flux distributions under pathological conditions.

This interpretation is consistent with independent single-nucleus transcriptomic studies reporting increased astrocyte abundance, upregulation of GFAP, and activation of signaling pathways associated with astrocyte reactivity across AD progression. Additionally, these studies have identified alterations in lipid and cholesterol metabolism, which align with the pathways highlighted in our models (Serrano-Pozo et al., 2024). However, this association remains speculative, as alternative explanations—including model-derived effects, transcriptional variability, or redistribution of metabolic fluxes—must also be considered (Cabezas et al., 2012; Zulfiqar et al., 2019).

At the subsystem level, the observed alterations suggest coordinated metabolic reorganization rather than isolated pathway disruptions. Changes in mitochondrial transport, fatty acid oxidation, cholesterol metabolism, and nucleotide interconversion indicate a shift in metabolic priorities associated with cellular stress and altered energetic demands. Importantly, these changes should be interpreted as relative redistributions within the constrained solution space of the model, rather than absolute increases or decreases in pathway activity.

At the reaction level, several patterns provide mechanistic insight into these system-wide changes. The marked increase in the r1666 reaction (Y + LAT2-mediated amino acid transport) during Early MCI may reflect a compensatory mechanism aimed at maintaining amino acid homeostasis under early proteotoxic stress (Horgusluoglu et al., 2022).

Similarly, the inversion of flux directionality observed in key glycolytic reactions (ENO, PGM) and mitochondrial transport (MALtm) suggests disruption of energetic homeostasis and redox balance (Bell and Mortiboys, 2026; Gollihue and Norris, 2020). These patterns are consistent with metabolic rewiring processes previously associated with astrocyte activation and stress adaptation, although within the context of expression-constrained modeling they represent predicted tendencies rather than direct biochemical evidence.

These reaction-level alterations can be interpreted in the context of astrocyte reactivity. While canonical markers such as GFAP, VIM, and STAT3 were not explicitly modeled, the observed metabolic patterns are consistent with functional programs associated with reactive astrocyte states across neurodegenerative conditions (Table 3). Nevertheless, this correspondence should be considered indirect and hypothesis-generating rather than definitive.

Additional metabolic changes further support a model of astrocytic reprogramming. Alterations in fatty acid activation reactions (FACOAL1832, FACOAL205) suggest dynamic lipid remodeling and a potential involvement of the APOE/ABCA1 axis in astrocyte dysfunction (G. Qi et al., 2021). In parallel, increased flux through nucleotide metabolism (CYTK6) and nuclear transport processes (UTPtn) may reflect activation of stress-responsive transcriptional programs (Haim et al., 2015). Dysregulation of polyamine transport (r1637) further supports the involvement of inflammatory metabolic axes, consistent with the role of polyamines in neuroinflammation and astrocyte functional states (Bhalla and Lee, 2025).

A key systems-level insight emerging from this analysis is the progressive loss of metabolic flexibility. Flux variability analysis revealed a narrowing of allowable flux ranges across disease stages, suggesting that astrocytes become increasingly constrained in their metabolic responses. This observation is consistent with emerging evidence that metabolic inflexibility represents an early and conserved feature of neurodegenerative processes (Shichkova et al., 2025). Notably, this reduction in flexibility is already detectable in Early MCI, supporting the idea that metabolic constraints arise early in disease progression.

From a functional perspective, these alterations have important implications for astrocyte-mediated processes. Disruption of glutamate–glutamine cycling may compromise neurotransmitter recycling and contribute to excitotoxicity, while alterations in redox-related pathways suggest impaired antioxidant capacity. Similarly, dysregulation of cholesterol metabolism and one-carbon pathways may affect membrane homeostasis, epigenetic regulation, and NADPH availability (Butterfield and Boyd-Kimball, 2018; Bailey and Gregory, 1999; Mauch et al., 2001; Field et al., 2014; Groot et al., 2024; Pfrieger, 2003).

A key limitation of the present modeling framework lies in the assumption of a proportional relationship between mRNA abundance and reaction capacity. Although this assumption enables the construction of condition-specific metabolic models, it does not account for the well-established decoupling between transcriptomic, proteomic, and metabolomic layers. mRNA abundance does not necessarily reflect protein levels due to post-transcriptional regulation, differences in translation efficiency, and protein turnover rates.

Moreover, enzymatic activity is influenced by multiple factors not captured by transcriptomic data alone, including post-translational modifications, substrate availability, cofactor dynamics, and allosteric regulation. As a result, transcriptome-constrained models should be interpreted as approximations of feasible metabolic states rather than direct representations of *in vivo* metabolic activity.

Therefore, the predicted flux distributions reflect potential metabolic configurations consistent with transcriptional constraints, but do not constitute direct measurements of enzymatic activity or intracellular metabolite levels.

Finally, it is important to recognize the limitations of the modeling framework. Expression-constrained flux balance analysis predicts feasible flux distributions rather than actual enzymatic rates or intracellular metabolite concentrations. Therefore, the observed metabolic alterations should be interpreted as hypothesis-generating and indicative of potential metabolic reprogramming, rather than as definitive causal mechanisms.

### 4.7 Loss of metabolic flexibility revealed by flux variability analysis

FVA revealed a progressive narrowing of allowable flux ranges across disease stages for key metabolites, including lactate, pyruvate, cholesterol, glutamine, methionine, glutathione, and hydrogen peroxide (Figure 4B). Rather than indicating precise intracellular concentrations, these results reflect a loss of metabolic flexibility, suggesting that astrocytes become increasingly constrained in their ability to respond to energetic and oxidative challenges (Le Douce et al., 2020; Virmani and Cirulli, 2022).

This early reduction in metabolic flexibility is consistent with emerging systems-level evidence suggesting that metabolic reprogramming precedes overt neurodegeneration. A recent study published by (Shichkova et al., 2025) that reports that progressive metabolic inflexibility represents a conserved early feature across neurodegenerative trajectories, supporting the view that reduced metabolic adaptability constitutes a primary vulnerability rather than a secondary consequence of neuronal loss. In this context, our astrocyte-specific models offer a possible cellular framework to interpret how such systemic metabolic rigidity may arise during prodromal stages of cognitive impairment.

Notably, reduced flux variability was observed not only in Advanced MCI and AD but also in Early MCI, implying that metabolic rigidity emerges early in the disease process. This convergence toward constrained flux spaces highlights a potentially shared vulnerability that may precede overt neurodegeneration and underscores the potential value of early metabolic interventions.

### 4.8 Disruption of neurotransmitter cycling and redox homeostasis

Astrocytes are central to the glutamate–glutamine cycle, and impairments in this pathway have been linked to excitotoxicity and cognitive decline. Model predictions indicated reduced flux capacity through glutamate and glutamine-related reactions as MCI progressed (Table 4). While constraint-based models do not directly estimate intracellular metabolite concentrations, altered exchange and internal reaction fluxes suggest compromised astrocytic support of neurotransmitter recycling.

### 4.9 Lipid and one-carbon metabolism as metabolic bottlenecks

**FIGURE 5:**
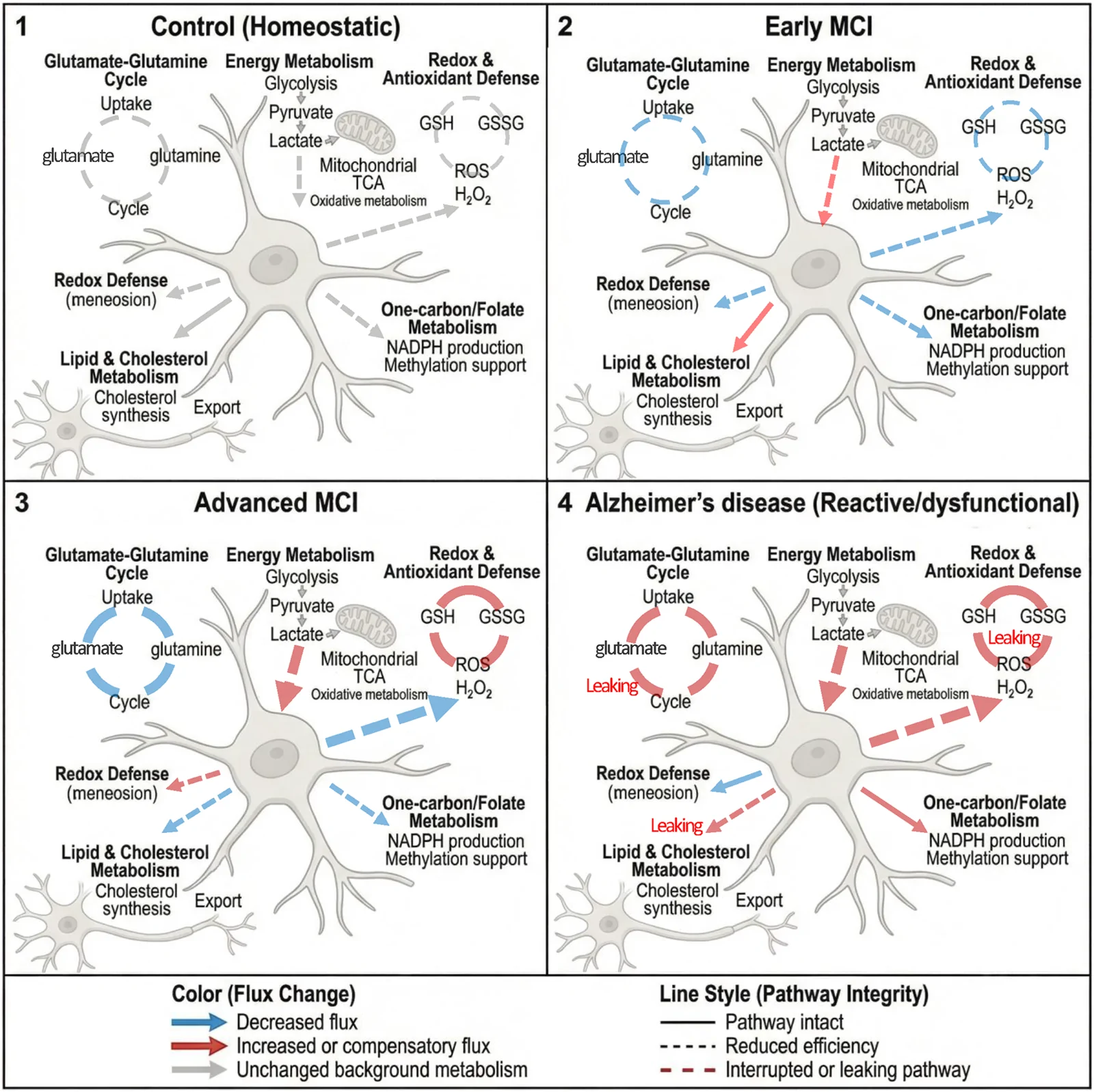
Schematic diagrams of the multi-omics metabolic model of astrocytes contextualized with transcriptomic data for three clinical conditions: (2) Early MCI, (3) Advanced MCI, and (4) AD. Metabolic pathways are color-coded based on the type of flux change observed: Light blue indicates decreased flux, red indicates increased flux, and yellow indicates complete inactivation of reactions. Progressive alterations are highlighted in key pathways such as glycolysis (reduced activity of enolase, GAPDH, and Triosephosphate Isomerase), glutamate–GABA metabolism, cholesterol synthesis (mevalonate pathway and 24-Dehydrocholesterol Reductase), and mitochondrial and lysosomal transport systems. AD exhibits a greater number of model-predicted inactivated or altered pathways, particularly in energy production, redox balance, and neurotransmission, reflecting the functional.

Cholesterol metabolism emerged as a sensitive pathway across disease stages (Figures 1–4, 5). Astrocytes are the primary source of cholesterol in the adult brain, and dysregulation of cholesterol synthesis and export -particularly via ATP-Binding Cassette Transporter A1-has been linked to synaptic dysfunction, tau pathology, and lysosomal impairment (Butterfield and Boyd-Kimball, 2018; Mauch et al., 2001; Groot et al., 2024; Pfrieger, 2003). Model predictions of altered cholesterol handling may be consistent with a convergence between compensatory metabolic responses and pathological lipid accumulation.

### 4.10 One-carbon metabolism and APOE-dependent metabolic rewiring

In parallel, one-carbon and folate metabolism pathways showed a progressive loss of activity, including reduced flux through Methylenetetrahydrofolate Dehydrogenase 1 (MTHFD1) and Methylenetetrahydrofolate Reductase (MTHFR) (Table 4). These changes may limit methylation capacity, nucleotide synthesis, and Nicotinamide Adenine Dinucleotide Phosphate (NADPH) availability, thereby compromising epigenetic regulation and antioxidant defense (Bailey and Gregory, 1999; Field et al., 2014).

The comparison between GEM-derived flux alterations and experimental observations from APOE4 astrocytes provides additional context for interpreting these metabolic changes. Partial concordance between computational predictions and isotope-resolved metabolomics data supports the biological relevance of the inferred metabolic rewiring, particularly in glutamate-centered metabolism and mitochondrial redox balance.

Within the glutamate metabolic module, the predicted reduction in glutaminase flux, together with experimentally observed increases in glutamate isotopologue labeling (Lysaker et al., 2025), suggests that glutamate metabolism is not simply downregulated but functionally reprogrammed. This reorganization likely reflects a redistribution of carbon flux across interconnected metabolic pathways, consistent with the central role of astrocytes in glutamate–glutamine cycling and neuronal metabolic support (Vandenberg and Ryan, 2013).

The inversion of flux direction observed in both NAD-dependent and NADP-dependent glutamate dehydrogenase reactions further supports the presence of metabolic rewiring. These reactions link amino acid metabolism with the TCA cycle and cellular redox balance, and their altered directionality suggests a decoupling between glutamate utilization and mitochondrial energy production. This is consistent with mitochondrial dysfunction and altered redox homeostasis described in APOE4 astrocytes (Dedic et al., 2018; Lysaker et al., 2025; Vandenberg and Ryan, 2013).

In contrast, the TCA cycle exhibited a more uniform reduction in flux magnitude, particularly in succinate-associated metabolism. This pattern aligns with experimental observations of reduced incorporation of glucose-derived carbon into TCA intermediates and impaired oxidative phosphorylation in APOE4 conditions (Lysaker et al., 2025).

Importantly, while the observed concordance supports the relevance of the modeling framework, discrepancies between datasets are expected due to fundamental differences between flux predictions and isotope-tracing measurements. Constraint-based models estimate feasible flux distributions under transcriptional constraints, whereas isotope-resolved metabolomics captures dynamic carbon flow and compartment-specific metabolic activity. Therefore, the agreement observed here should be interpreted as functional convergence rather than direct quantitative equivalence.

### 4.11 Limitations

Collectively, our results suggest that astrocyte metabolic reprogramming during MCI and AD is a coordinated, stage-dependent process involving neurotransmitter cycling, lipid handling, redox regulation, and biosynthetic capacity. Rather than being solely passive consequences of neuronal degeneration, these alterations may also contribute to disease progression.

Nevertheless, several limitations warrant consideration. The use of microarray-derived pseudo-counts and deconvolution-based signatures introduces uncertainty in absolute expression levels. Flux predictions are constrained by transcriptional data and therefore do not fully capture the multi-layered regulation of cellular metabolism. A well-established gap exists between transcriptomic, proteomic, and metabolomic levels: mRNA abundance does not necessarily correlate with protein levels due to post-transcriptional regulation, differential translation efficiency, and protein turnover. Moreover, enzymatic activity is further modulated by post-translational modifications, substrate availability, cofactor dynamics, and allosteric regulation. Consequently, the inferred flux distributions should be interpreted as feasible metabolic states constrained by gene expression, rather than direct measurements of enzymatic activity or metabolite concentrations.

In addition, the astrocyte-associated expression profiles were inferred from bulk transcriptomic data under deconvolution assumptions and are therefore subject to uncertainty arising from latent component estimation, overlap among related cell populations, and transformation-related noise. Consequently, the inferred stage-dependent astrocytic differences should be interpreted as robust model-supported trends rather than direct cell-resolved measurements.

While the biomass objective was reformulated to approximate astrocyte maintenance demands, it remains a simplified representation and does not fully capture the specialized physiological roles of astrocytes *in vivo*. Alternative objective functions, such as ATP production, redox balance, or neurotransmitter cycling, may provide complementary insights and should be explored in future studies.

Additionally, the modeling framework does not allow direct statistical testing of differences between conditions, as flux balance analysis produces deterministic solutions rather than distributions derived from biological replicates. Consequently, the observed differences should be interpreted as consistent model-derived trends rather than statistically validated effects. Future studies incorporating flux sampling approaches (e.g., Monte Carlo sampling of the solution space) could provide distributions of feasible fluxes and enable statistical comparison across conditions.

Although the integration of transcriptomic data with constraint-based metabolic modeling provides a framework to infer functional metabolic states, this study does not constitute direct experimental validation of metabolic fluxes *in vivo*. The agreement observed with independent datasets should be interpreted as cross-modal functional convergence. The inferred metabolic alterations represent plausible, model-supported hypotheses consistent with existing evidence, but require further validation through targeted metabolomic and fluxomic studies. Despite these limitations, this study provides an integrative framework linking transcriptomic alterations to astrocyte-specific metabolic dysfunction across the MCI–AD continuum. Future work incorporating proteomics, metabolomics, and refined astrocyte reconstructions will be essential to validate these predictions and assess their translational relevance.

## 5 Conclusion

This study integrates unsupervised CDSeq with genome-scale metabolic modeling to build stage-resolved, astrocyte-specific metabolic models (control, early MCI, advanced MCI, and AD) from hippocampal CA1 data, enabling a mechanistic translation of gene-expression shifts into predicted metabolic consequences. Overall, the results support a predicted early, stage-dependent metabolic reprogramming of astrocytes consistent with disease-associated metabolic alterations along the MCI–AD continuum, characterized by reduced metabolic flexibility and disruptions in core neuronal-support pathways, including redox homeostasis (glutathione), glutamate–glutamine cycling, energy metabolism (glycolysis/pyruvate), lipid/cholesterol handling, and one-carbon/folate metabolism. The apparent partial rebound of the biomass-derived maintenance proxy in AD may reflect a compensatory shift that is consistent with reactive-like astrocyte states, rather than true functional recovery. While these predictions require further experimental validation and would benefit from integration with additional omics layers, the proposed framework provides a reproducible strategy to identify early astrocyte metabolic vulnerabilities and prioritize pathways and targets for future validation in strategies aimed at understanding or potentially modifying progression to dementia.

## Data Availability

Publicly available datasets were analyzed in this study. This data can be found here: Transcriptomic data analyzed in this study are publicly available in the Gene Expression Omnibus (GEO) under accession number GSE28146 (https://www.ncbi.nlm.nih.gov/geo/query/acc.cgi?acc&equals;GSE28146). The reconstructed metabolic models and analysis scripts are openly available at: https://github.com/mangaritar/Astrocyte-metabolic-modeling-MCI-AD.
