# Correction: Transcriptome-informed metabolic modeling reveals astrocyte-specific vulnerabilities in mild cognitive impairment and Alzheimer’s disease progression

**DOI:** 10.3389/fbinf.2026.1953979

**Published:** 2026-08-11

**Authors:** Andrea Angarita-Rodríguez, Viviana Vargas-López, Andrés Pinzón, Adrián Sandoval-Hernandez, Kai Kang, Leping Li, Jason Papin, Pedro Puentes-Rozo, Andrés Felipe Aristizábal, Janneth González

**Affiliations:** 1 Departamento de Nutrición y Bioquímica, Facultad de Ciencias, Pontificia Universidad Javeriana, Bogotá, Colombia; 2 Laboratorio de Bioinformática y Biología de Sistemas, Universidad Nacional de Colombia Bogotá, Bogotá, Colombia; 3 Biostatistics and Computational Biology Branch at NIEHS, Bethesda, MD, United States; 4 Grupo de Neurociencias y Muerte Celular, Instituto de Genética, Universidad Nacional de Colombia, Bogotá, Colombia; 5 Departamento de Química, Facultad de Ciencias, Universidad Nacional de Colombia, Bogotá, Colombia; 6 Department of Mathematics and Statistics, University of North Carolina Wilmington, Wilmington, NC, United States; 7 Department of Biomedical Engineering, University of Virginia, Charlottesville, VA, United States; 8 Department of Medicine, Division of Infectious Diseases and International Health, University of Virginia, Charlottesville, VA, United States; 9 Department of Biochemistry and Molecular Genetics, University of Virginia, Charlottesville, VA, United States; 10 Grupo de Neurociencias del Caribe, Unidad de Neurociencias Cognitivas, Universidad Simón Bolívar, Barranquilla, Colombia; 11 Grupo de Neurociencias del Caribe, Universidad del Atlántico, Barranquilla, Colombia

**Keywords:** genome-scale metabolic models, flux balance analysis, metabolic reprogramming, transcriptome, deconvolution, mild cognitive impairment, Alzheimer’s disease, astrocyte

In the published article, the Funding statement was published incorrectly. The article stated:

“The author(s) declared that financial support was not received for this work and/or its publication.”

The correct Funding statement is:

“This work was supported by the Sistema General de Regalías (SGR), Bogotá, Colombia, under grant BPIN 2020000100357.”

The original version of this article has been updated.

