# Supplementary material for: Transcriptome-informed metabolic modeling reveals astrocyte-specific vulnerabilities in mild cognitive impairment and Alzheimer’s disease progression
Source: Front Bioinform. 2026 Jun 2;6:1816121. doi: 10.3389/fbinf.2026.1816121 (PMC13269292; doi:10.3389/fbinf.2026.1816121)
Supplement: Supplementary file 2 [file Supplementaryfile1.docx]

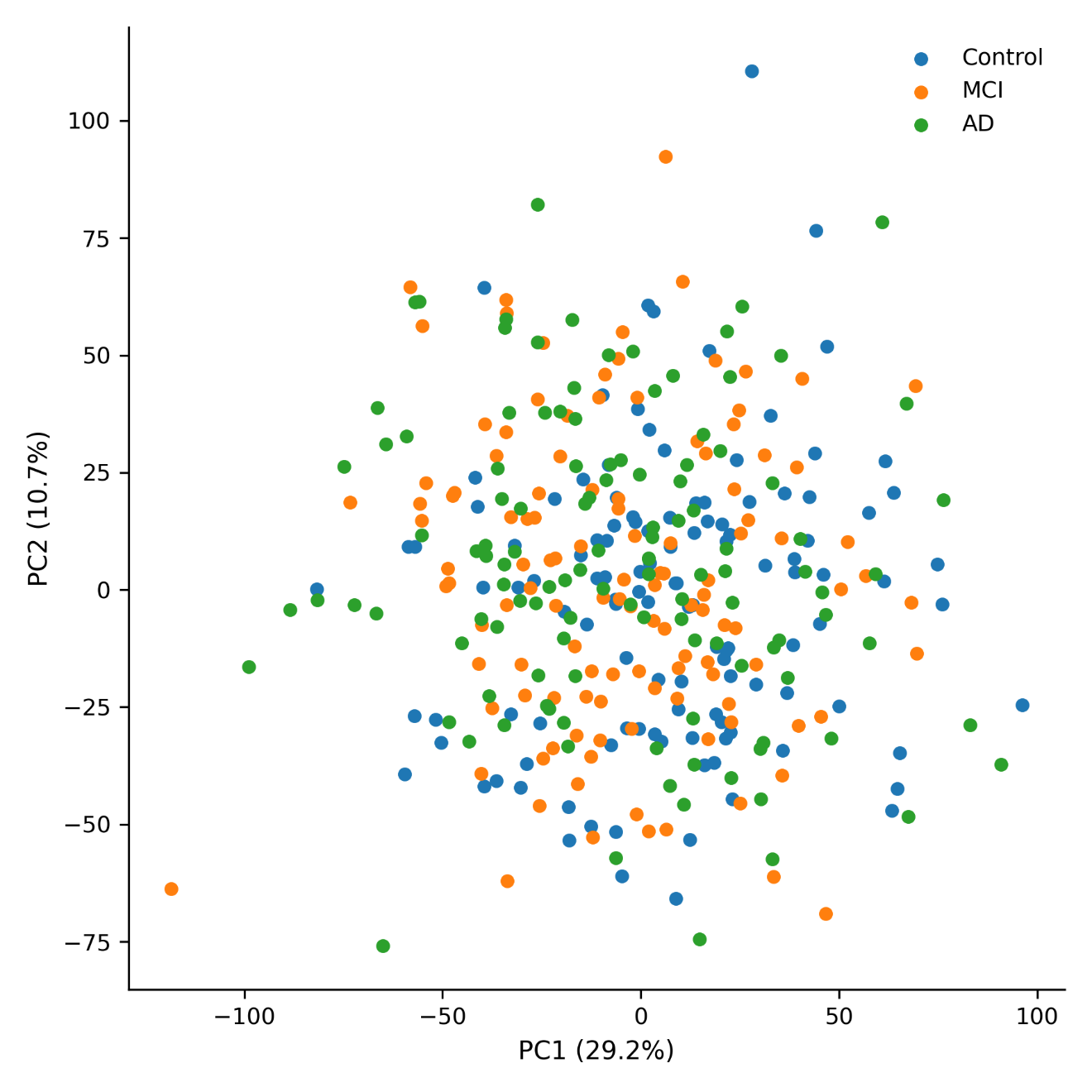


**Supplementary figure 1.** Principal Component Analysis (PCA) corrected for batch effects showing the distribution of samples from the GSE63060 dataset from peripheral blood samples in control, mild cognitive impairment or AD patients. Samples are grouped according to clinical classification based on Mini-Mental State Examination (MMSE) scores: Control (green, MMSE ≥ 25), MCI-Incipient (yellow, MMSE 20–26), MCI-Moderate (orange, MMSE 14–19), MCI-Severe (red, MMSE ≤ 14), and Alzheimer's disease (purple, with additional neuropathological confirmation).

**
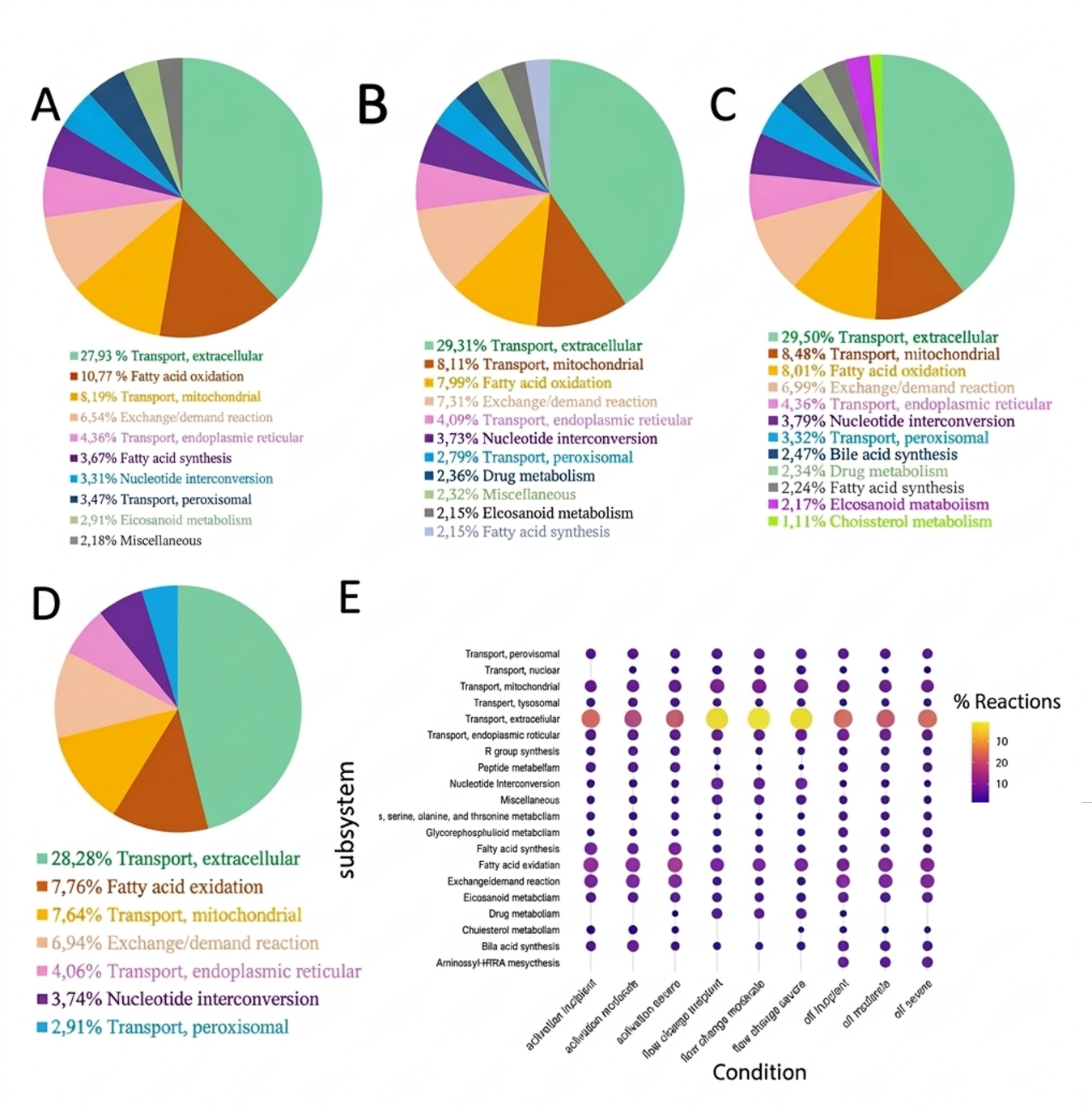
**

**Supplementary figure 2. Metabolic Phenotype, Distribution and functional enrichment of metabolic reactions by subsystem in astrocytes across clinical conditions. (A–D)** Pie charts showing the percentage distribution of active metabolic reactions grouped by functional subsystem in astrocyte models under control (A), Early MCI (B), Advanced MCI (C), and AD (D) clinical conditions. A consistently high proportion of reactions is associated with extracellular transport across all conditions, along with progressive changes in subsystems such as fatty acid oxidation, mitochondrial transport, bile acid synthesis, and cholesterol metabolism. (E) Bubble plot summarizing the relative contribution of each subsystem across clinical stages. Color intensity represents the relative percentage of reactions in each subsystem, while bubble size indicates the absolute percentage of reactions. Notable shifts are observed in pathways such as peroxisomal and mitochondrial transport, lipid metabolism, fatty acid synthesis, endoplasmic reticulum transport, and exchange reactions, showing stepwise increases or decreases throughout the progression of cognitive impairment.


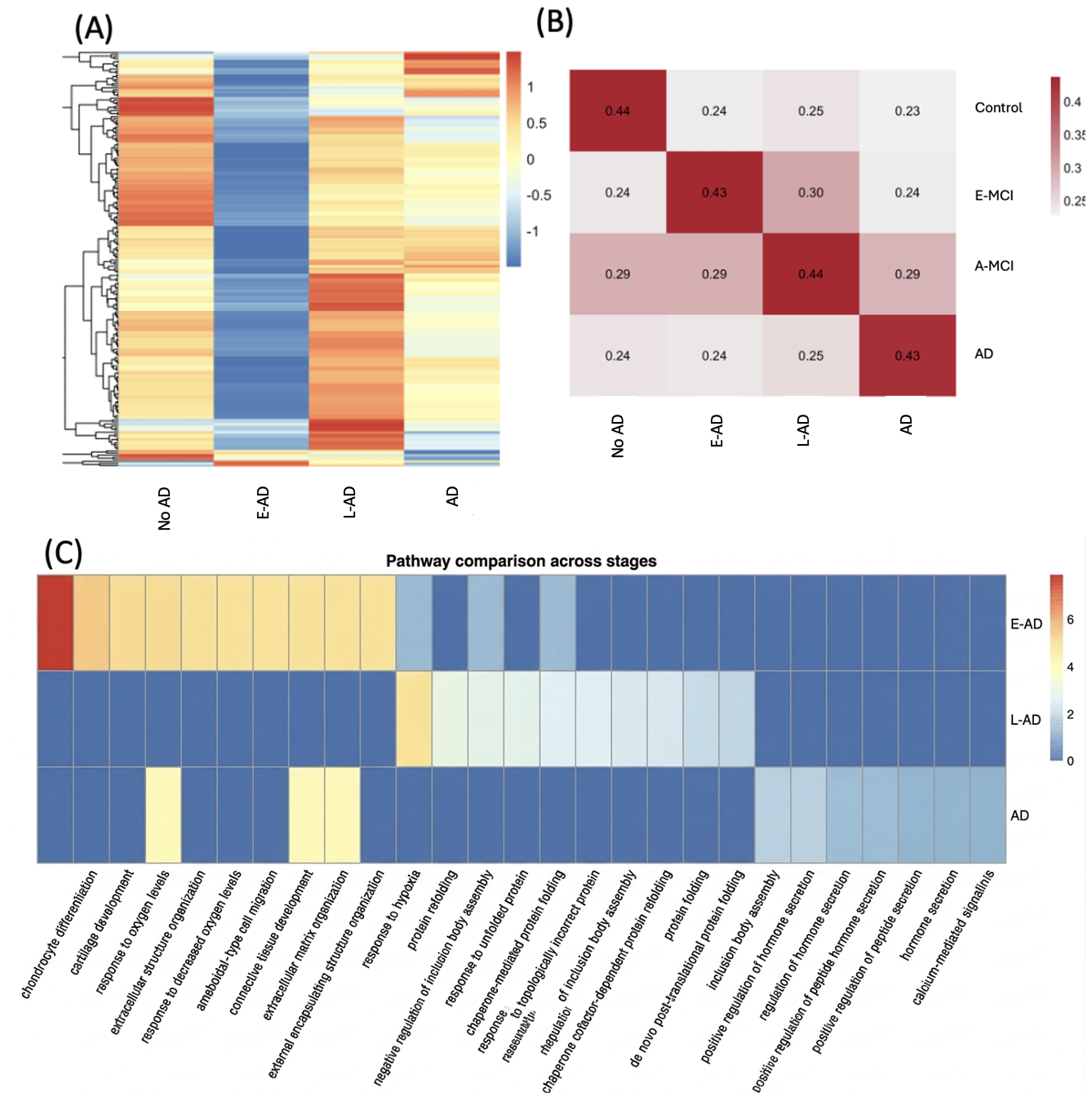


**Supplementary figure 3. Stage-dependent transcriptional and functional alterations across disease progression.** (A) Heatmap of gene expression dynamics across disease stages (E-AD, L-AD, and AD). Rows represent genes and columns correspond to disease stages. Values are scaled expression levels, highlighting coordinated patterns of upregulation and downregulation across progression. (B) Correlation matrix between stages based on gene expression profiles. Each cell represents the pairwise similarity between stages, showing higher within-stage consistency and gradual divergence across disease progression. (C) Functional enrichment heatmap of significantly enriched biological processes derived from stage-specific gene sets. Color intensity represents the enrichment score (log adjusted p-value), with warmer colors indicating stronger enrichment. Distinct functional modules emerge across stages, reflecting early adaptive responses (E-AD), intermediate remodeling (L-AD), and late-stage dysregulation (AD).
