# Supplementary material for: Transcriptome-informed metabolic modeling reveals astrocyte-specific vulnerabilities in mild cognitive impairment and Alzheimer’s disease progression
Source: Front Bioinform. 2026 Jun 2;6:1816121. doi: 10.3389/fbinf.2026.1816121 (PMC13269292; doi:10.3389/fbinf.2026.1816121)
Supplement: Supplementary file 5 [file Table6.docx]

**Supplementary Table 2. Metabolic reactions and pathways modulated across stages of Mild Cognitive Impairment (MCI): simulated fluxes (mmol/gDW/hr).** This table presents a curated list of key metabolic pathways and their associated enzymatic reactions potentially involved in the progression of Mild Cognitive Impairment (MCI). The Pathway column indicates the metabolic subsystem (e.g., Glycolysis, Glutamate-GABA cycle, Methionine metabolism) to which each reaction belongs. RxnID refers to the reaction identifier from the genome-scale metabolic model. The Name column specifies the enzyme or process catalyzing the reaction. The numerical columns (Control, Incipient, Moderate, Severe) report the simulated reaction fluxes under healthy and progressively impaired conditions, based on gene expression contextualization or flux balance analysis. All flux values are expressed in millimoles per gram of dry weight per hour (mmol/gDW/hr).

| **Pathway** | **RxnID** | **Name** | **Control** | **Incipient** | **Moderate** | **Severe** |
| --- | --- | --- | --- | --- | --- | --- |
| Glycolysis | CBPPer | Copper (Cu²⁺) ABC transporter | 0,005944 | 0,006524 | 0,011367 | 0,008269 |
| Glycolysis | r0165 |  | 0 | 0 | 2,604305 | 0 |
| Glycolysis | r0173 | (S)-Lactate:NAD⁺ oxidoreductase (Lactate ofhydrogenase) | 4,1819 | 0,58275 | 0,219721 | -0,321 |
| Glycolysis | r0202 | Sn-Glycerol-3-phosphate:NAD⁺ 2-oxidoreductase | -3,0042 | 0,777 | -0,52686 | -0,723 |
| Glycolysis | ENO | Enolase | -9,837 | 0,777 | 0,637 | 0,723 |
| Glycolysis | GAPD | Gliceralofhído-3-phosphate ofhydrogenase | 0 | 0,346006 | 0,161094 | 0,723 |
| Glycolysis | PGI | Glucose-6-phosphate isomerase | -3,2552 | -4,65111 | -3,5215 | -4,17204 |
| Glycolysis | PGM | Phosphoglicerato mutase | 9,8375 | -0,777 | -0,637 | -0,723 |
| Glycolysis | PGMT | Phosphoglucomutase | 2,8027 | 0,777 | 0,637 | 0,723 |
| Glycolysis | TPI | Triosa-phosphate isomerase | 7,3834 | -0,43201 | -0,43821 | -0,19276 |
| Glycolysis | LDH_L | L-Lactate ofhydrogenase | 5,0361 | 0,102685 | -0,637 | -0,63933 |
| Glycolysis | PYK | Pyruvate kinase | 2,4213 | -0,01205 | 0 | 0 |
| Glycolysis | HMR_7748 | Beta-D-Glucose-6-phosphate cetol-isomerase | -4,4697 | -4,64754 | -3,51516 | -4,1661 |
| Glycolysis | HMR_7749 | Beta-D-Glucose-6-phosphate cetol-isomerase | 4,4697 | 4,6618 | 3,517084 | 4,1976 |
| Glycolysis | EX_lac_L[e] | Intercambio of L-lactato (extracelular) | -9,98298 | -2,02609 | -0,75354 | -1,20867 |
| Beta-Oxidation | HMR_0200 | Ligasa of acil-CoA of ácidos grasos of caofna larga | -4,49109 | -0,12377 | 2,26E-21 | 3,77E-22 |
| Beta-Oxidation | HMR_0267 | Ligasa of acil-CoA of ácidos grasos of caofna larga | 1,0387 | 0,777 | 0,637 | 0,723 |
| Beta-Oxidation | HMR_0433 | Ligasa of acil-CoA of ácidos grasos of caofna larga | -3,1E-18 | 0 | 2,26E-21 | 3,77E-22 |
| Beta-Oxidation | HMR_2608 | Carnitina O-palmitoiltransferasa | -0,07439 | 0 | -8,5E-06 | 0 |
| Beta-Oxidation | HMR_2611 | Acil-CoA ofhydrogenase of caofna larga | -4,49109 | -0,12377 | -9,2E-22 | 4,59E-22 |
| Beta-Oxidation | HMR_2666 | Hidratasa of enoil-CoA of caofna larga | 1,0387 | 0,777 | 0,637 | 0,723 |
| Glutamate-GABA | GLUDxm | Glutamate ofhydrogenase (NAD⁺), mitocondrial | 5,0361 | -0,777 | 0,637 | 0,643245 |
| Glutamate-GABA | GLUDym | Glutamate ofhydrogenase (NADP⁺), mitocondrial | -5,0361 | 0,699337 | -0,637 | -0,723 |
| Glutamate-GABA | ABTArm | Transaminasa of 4-aminobutirato (GABA transaminasa) | -1,27459 | -0,41636 | -0,25679 | -0,723 |
| Glutamate-GABA | ALATA_L | Alanina transaminasa (ALT) | -5,0361 | -0,777 | -0,637 | -0,723 |
| Glutamate-GABA | r0178 | S-aofnosylmethionine ofcarboxylase | -2,7801 | -1,19336 | -0,89379 | -0,723 |
| Glutamate-GABA | r0179 | Succinato semialofhído:NADP⁺ oxidoreductase | 1,505515 | 0,777 | 0,637 | 0 |
| Glutamate-GABA | r0464 |  | -5,0361 | 0,777 | -0,637 | 0,723 |
| Glutamate-GABA | r0549 | ATP:riboflavina 5'-fosfotransferasa | 5,0361 | -0,777 | 0,637 | -0,723 |
| Glutamate-GABA | r0129 |  | 7,86 | 0,777 | 0,637 | 0,723 |
| Glutamate-GABA | r0130 | 4-Hidroxibenzoil-CoA hidrolasa | 1,616748 | 0,777 | 0,637 | 0,723 |
| Glutamate-GABA | r0021 | L-Glutamate 1-carboxilasa (formadora of 4-aminobutanoato) | 2,7695 | 0,73571 | 0,60315 | -0,29882 |
| Methionine | EX_met_L[e] | Intercambio of L-metionina (extracelular) | -0,03 | -0,03 | -0,03 | -0,03 |
| Methionine | HMR_5143 | | 0 | -0,0032 | -0,00052 | -0,09115 |
| Methionine | CYSGLTH | Síntesis of glutatión a partir of cisteína y glutamato | -5,0361 | -0,777 | -0,637 | 0,2604 |
| Methionine | r0027 | Glutatión:peróxido of hidrógeno oxidoreductase | -5,0361 | -0,777 | -0,637 | -0,723 |
| Methionine | HMR_4701 | | 0,683325 | 0,039188 | -0,00876 | 0,009862 |
| Cholesterol | ACACT1x | Acetil-CoA acetiltransferasa (tiolasa) | 7,913 | 0,777 | 0,637 | 0,723 |
| Cholesterol | r0463 | Hidroximetilglutaril-CoA synthase | -7,913 | -0,777 | -0,637 | -0,723 |
| Cholesterol | r1380 | Reductasa of 24-ofhidrocolesterol | -9,858 | 0 | -0,00475 | -0,723 |
| Cholesterol | DHCR241r | Reductasa of 24-ofhidrocolesterol | 9,858 | 0 | 0,004752 | 0,723 |
| Cholesterol | P450SCC1m | Colesterol monooxigenasa (P450scc) | -0,55534 | -0,777 | -0,637 | -0,723 |
| Cholesterol | EX_chsterol[e] | Intercambio of colesterol (extracelular) | -1,1 | -0,00661 | -0,00554 | -0,00623 |
| Lysosomes | ADNtl | Transporte facilitado of aofnosina ofsof el lisosoma | 1,171 | 0,185168 | 0,152703 | 0,172527 |
| Lysosomes | GALGLUSIDEtl | Transporte of galactoglucósido al lisosoma | -5,0361 | -0,777 | -0,637 | 0 |
| Lysosomes | H2O2tly | Transporte of peróxido of hidrógeno al lisosoma | -2,31072 | 0,481084 | 0,479408 | 0,723 |
| Lysosomes | H2Otly | Transporte of agua al lisosoma | 5,0361 | -0,777 | -0,637 | -0,723 |
| Lysosomes | r0932 | 1,1,2-Trichloroethylene to trans-1,2-Dichloroethene | 0 | -0,777 | 0,010297 | -0,723 |
| Lysosomes | r0986 | 3-hydroxy-2,6-dimethyl-5-methylene-heptanoyl-CoA ofhydrogenase | 5,0361 | 0,777 | 0,637 | 0,723 |
| Lysosomes | r0987 | Vesicular Transport | -5,0361 | -0,777 | -0,637 | -0,723 |
| Lysosomes | r1052 | Vesicular Transport | 3,485534 | 0,777 | 0,637 | 0,723 |
| Lysosomes | r1301 | Vesicular Transport | 0 | 0,777 | 0,637 | 0,723 |
| Lysosomes | r1302 | Vesicular Transport | 0 | -0,777 | -0,637 | -0,723 |
| Lysosomes | r1304 | Vesicular Transport | 5,0361 | 0,777 | 0,637 | 0,723 |
| Lysosomes | r1364 | Vesicular Transport | 9,343 | 0,777 | 0 | 0,723 |
| Lysosomes | r1365 | Vesicular Transport | 9,343 | 0,777 | 0 | 0,723 |
| Lysosomes | r1150 | Facilitated Diffusion | 4,146549 | 0,185168 | 0,321815 | 0,172527 |
| Lysosomes | NO2te | Nitrite Dioxiof Transport, Assumed Diffusion | 5,0361 | -0,777 | -0,637 | -0,723 |
| Lysosomes | HMR_2087 | HMR_2087 | -2,31072 | 0,481084 | 0,479408 | 0,723 |
| Lysosomes | HMR_2090 | HMR_2090 | 2,310723 | -0,48108 | -0,47941 | -0,723 |
| Lysosomes | HMR_3597 | HMR_3597 | 9,89602 | 0,777 | 0,637 | 0,723 |
| Lysosomes | HMR_7199 | HMR_7199 | -5,0361 | -0,777 | -0,637 | -0,723 |
| Lysosomes | HMR_7711 | HMR_7711 | 1,171 | 0,185168 | 0,152703 | 0,172527 |
| Tau | PI45P5Pn | Fosfatidilinositol-4,5-bisphosphate 5-fosfatasa nuclear | 0 | 0,777 | 0,637 | 0 |
| Tau | GNMT | Glicina N-metiltransferasa | 0 | 0,068903 | -0,02159 | 0 |
| Tau | r0160 | Serina-piruvato aminotransferasa | 0 | 0,430994 | 0,489869 | 0,48567 |
| Tau | GHMT2r | Glicina hidroximetiltransferasa, reversible | 3,015 | 0,777 | 0,637 | 0,723 |
| Tau | RE2427M | Glicina N-aciltransferasa | 1,68607 | 0,777 | 0,637 | 0,723 |
| Tau | ATPS4mi | ATP synthase (complejo V mitocondrial) | 5,0361 | 0,777 | 0,637 | 0,723 |
| Tau | NADH2_u10mi | NADH ofhydrogenase (complejo I mitocondrial) | 5,0361 | 0,106674 | 0,051592 | 0 |
| Tau | CYOOm2i | Citocromo c oxidasa (complejo IV mitocondrial) | -0,24584 | 0,670326 | 0,585408 | 0,723 |
| Lactate | LDH_L | L-lactato ofhydrogenase | 5,0361 | 0,102685 | -0,637 | -0,63933 |
| Lactate | EX_lac_L[e] | Intercambio of L-lactato (extracelular) | -9,982 | -2,026 | -0,75354 | -1,2086 |
| Glutamine | HMR_9802 | Glutaminesa | 6,8274 | 0 | 0 | 0,897609 |
| Glutamine | EX_gln_L[e] | Intercambio of L-glutamina (extracelular) | -1,84498 | -2,95918 | -0,84937 | -4,51826 |
| Glutathione | r0021 | L-glutamato ofscarboxilasa (formadora of GABA) | 2,7695 | 0,73571 | 0,60315 | -0,29882 |
| Glutathione | r0027 | Glutatión:peróxido of hidrógeno oxidoreductase | -5,0361 | -0,777 | -0,637 | -0,723 |
| Glutathione | CYSGLTH | Síntesis of glutatión a partir of cisteína y glutamato | -5,0361 | -0,777 | -0,637 | 0,2604 |
